# Influence of Pipe Material and Liquid Properties on Water-Hammer Wave Propagation and Transient Pressure Response

**DOI:** 10.3390/ma19214574

**Published:** 2026-10-29

**Authors:** Kamil Urbanowicz, Adam Deptuła, Marcin Chodźko, Łukasz Blacha, Armin Alavi, Olga Orynycz

**Affiliations:** 1 Faculty of Mechanical Engineering and Mechatronics, West Pomeranian University of Technology in Szczecin, 70-310 Szczecin, Poland; marcin.chodzko@zut.edu.pl (M.C.); as61302@zut.edu.pl (A.A.); 2 Faculty of Production Engineering and Logistics, Opole University of Technology, 45-758 Opole, Poland; a.deptula@po.edu.pl; 3 Faculty of Mechanical Engineering, Opole University of Technology, 45-271 Opole, Poland; l.blacha@po.edu.pl; 4 Faculty of Engineering Management, Bialystok University of Technology, Wiejska Street 45A, 15-351 Bialystok, Poland; o.orynycz@pb.edu.pl

**Keywords:** pipe material properties, fluid–structure interaction, sensitivity analysis, pressurized pipelines, method of characteristics, water hammer modeling

## Abstract

Water-hammer transients in pressurized pipelines are governed by the coupled compressibility of the liquid and the elastic response of the pipe wall. This study quantifies the sensitivity of transient pressure responses to variations in pipe material, system geometry, and liquid properties using a one-dimensional Method of Characteristics (MOC) model incorporating a convolution-based formulation of unsteady friction. The model is applied to an experimental copper-pipe system under laminar and turbulent flow conditions. A one-factor-at-a-time (OFAT) sensitivity analysis is performed by independently varying selected parameters by ±5% and ±10%, including Young’s modulus, Poisson’s ratio, pipe-wall thickness, internal radius, liquid density, bulk modulus, and kinematic viscosity. The results show that liquid density, liquid bulk modulus, and pipe Young’s modulus significantly affect pressure-wave celerity and the temporal evolution of the transient response. Pipe internal radius has a pronounced influence on the pressure response, while the effects of viscosity and bulk modulus become more significant under turbulent-flow conditions. Combined variations in material, pipeline geometry, and liquid properties yield pressure-wave celerities ranging from approximately 1159 to 1479 m/s, demonstrating the substantial influence of input-property uncertainty on predicted transient behavior. The OFAT results are further analyzed using multiple-valued logic (MVL) decision trees and classification and regression tree (CART) models to identify dominant parameter–response relationships and threshold values. The findings highlight the importance of accurately characterizing pipe-material and liquid properties for reliable water-hammer prediction and the assessment of pressurized pipeline systems.

## 1. Introduction

The mechanical properties of pipeline materials are fundamental to the propagation of pressure disturbances in liquid-filled conduits. During a water-hammer event, pressure wave propagation is governed not only by the compressibility of the transported liquid but also by the elastic deformation of the pipe wall. Consequently, Young’s modulus, Poisson’s ratio, pipe-wall thickness, pipe diameter, and support conditions influence the pressure-wave celerity and, through it, the temporal structure of the transient response. The contribution of the liquid is determined primarily by its density and bulk modulus, whereas its viscosity influences the dissipative component of the transient response. These effects are coupled through the governing equations; therefore, uncertainties in either material or liquid properties can affect the predicted pressure amplitudes, wave arrival times, and attenuation rates.

Recent reviews have emphasized that water-hammer wave propagation results from the coupling between liquid compressibility and pipe-wall deformation, while wave attenuation depends on additional mechanisms associated with unsteady flow and the mechanical response of the pipe wall. Plouraboué [1] reviewed the theoretical foundations of water-hammer waves, including fluid–structure interaction, dissipation mechanisms, and the influence of viscoelastic pipe-wall behavior. These findings underline the need to distinguish the effects of liquid properties on wave propagation from those associated with pipe material and structural response.

Metal pipes are still widely used in high-pressure hydraulic systems, district-heating installations, nuclear reactor cooling systems, and long-distance transmission pipelines. In such pipes, pressure wave celerities are approximately 3 to 4 times higher than those in plastic pipes. Consequently, the pressure rise during a water hammer event can be calculated using the Joukowsky equation [2]:

∆*p* = *cρ*∆*v* (1)

where *c* is the pressure wave celerity [m/s]; *ρ* is the liquid density [kg/m^3^] and Δ*v* is the change in velocity [m/s]. For comparable flow conditions, the pressure rise in a metal pipe is around three times that in an analogous plastic pipe. Moreover, because metal pipe walls deform elastically, whereas plastic pipe walls exhibit viscoelastic behavior [3], the maximum pressure may be up to twice the value calculated using Equation (1). Such an increase can result from the superposition of primary and secondary pressure waves during transient flows involving vapor-cavity formation [4,5].

Recent experimental and numerical investigations have provided further evidence of the influence of pipe material on water-hammer characteristics. Kandil et al. [6] investigated five pipe materials and showed that differences in material stiffness and viscoelastic behavior affect transient pressure signals and their frequency content. Their results also demonstrated discrepancies between experimentally measured and numerically predicted water-hammer frequencies, highlighting the importance of representing the actual mechanical behavior and boundary conditions of the pipeline. More recently, Kadi-Rouabah et al. [7] compared rigid and viscoelastic pipelines using the Method of Characteristics and sensitivity analysis. Their results identified pipe material as the most influential factor in the maximum pressure head among the factors considered, while both pipe material and valve-closure law contributed to the damping response.

Also, pipe vibrations play a significant role in modeling these systems [8,9,10]. However, pipe vibrations are not considered in the present study because of the additional modeling complexity. Hydraulic systems are protected against harmful pressure surges by using protective devices such as air chambers, surge tanks, air vents, safety valves, and check valves [11,12]. To reduce the cost of protective devices, slow-closing valves should be used whenever appropriate, as they can help limit the formation of high-pressure surges during water hammer events.

System performance can also be improved at the design stage by selecting appropriate values of the parameters describing the pipe and its support conditions (*L*, *E*_0_, *R*, *e*, *α*, *Ξ*) as well as properties of the working fluid (*ρ*, *K*, *ν*). Water hammer should not only be regarded solely as a phenomenon to be mitigated, but its benefits are sometimes harnessed to improve the operation of hydraulic rams [13] and devices that generate mechanical vibrations [14]. In the present study, a sensitivity analysis based on multiple-valued logic (MVL) trees was used to investigate the influence of individual parameters on transient flow behavior. Unlike binary approaches, MVL allows parameters to take multiple discrete or graded values, making the method suitable for representing engineering tolerances, parameter classes, and regime-dependent behavior. This enables the derivation of compact, interpretable decision rules that describe both the magnitude and direction of each parameter’s influence.

Recent studies have also extended water-hammer sensitivity analysis beyond conventional parameter-by-parameter comparisons. Zha et al. [15] developed a reliability-based sensitivity framework for water-hammer-induced pipe failure, incorporating parameter uncertainty and fluid–structure interaction through a Kriging-based approach. Their findings demonstrated that input variables can have different effects on the nonlinear structural response and reliability of the pipeline. In a different approach, Kadi-Rouabah et al. [7] used analysis of variance to quantify the contributions of pipe material and valve-operation parameters to pressure extremes and damping. These studies demonstrate the value of systematic sensitivity assessment, but they employ approaches different from the combined one-factor-at-a-time (OFAT), MVL decision-tree, and classification and regression tree (CART) regression framework adopted in the present study.

The originality of the present study lies in combining OFAT sensitivity analysis, MVL decision structures, and CART models to investigate water-hammer responses. The OFAT analysis quantifies and ranks the influence of individual parameters on the transient response, while the MVL decision structures express the identified relationships as interpretable rules. The CART models provide a complementary data-driven approach for identifying parameter–response relationships and threshold values.

The proposed framework is applied separately to laminar and turbulent flow conditions to compare the dominant parameters influencing pressure-wave propagation and transient pressure responses. By integrating quantitative sensitivity analysis with interpretable decision rules and regression-tree models, the study provides a consistent framework for assessing the effects of pipe material, system geometry, and liquid properties on water-hammer behavior. The analysis is restricted to individual parameter variations and does not evaluate interactions between parameters varied simultaneously.

## 2. Basic Equations Describing Transient Flow

The equations of continuity and motion that represent the transient flow have the form:

(2) $$\left\{\begin{array}{c} \frac{\partial p}{\partial t}+\rho c^{2}\frac{\partial v}{\partial x}=0\\ \rho \frac{\partial v}{\partial t}+\frac{\partial p}{\partial x}+\frac{2\rho }{R}\left(\frac{v\left|v\right|}{8}f+\frac{2\nu }{R}\int_{0}^{t}\frac{\partial v}{\partial t}\left(u\right)\cdot w\left(t-u\right)\mathrm{du}\right)=0 \end{array}\right.$$

where *p*—pressure [Pa]; *t*—time [s]; *x*—distance along the pipe [m]; *v*—velocity [m/s]; *R*—inner pipe radius [m]; *f*—friction coefficient [-]; *ν*—kinematic viscosity [m^2^/s]; and *w*(*t* − *u*)—weighting function [-].

The pressure wave speed *c* = *f*(*ρ*, *K*, *ν_P_*, *R*, *E*_0_, *e*):

(3) $$c=\sqrt{\frac{1}{\rho \left[\frac{1}{K}+\frac{\Xi }{E_{0}}\right]}},$$

where $\Xi =\frac{D}{e}\alpha$ [-]—enhanced parameter *α*, *D* = 2*R*—inner pipe diameter [m], *K*—liquid bulk modulus [Pa], *E*_0_—pipe elastic modulus [Pa], *e*—thickness of pipe wall [m], *α*—parameter reporting support condition of pipe [-].

As can be seen from Equation (3), in single-phase flow, the pressure wave propagation velocity is a function of six parameters. Some of these parameters appear during hydraulic shock modeling only in the formula for the pressure wave propagation velocity; these are *K* and *E*_0_. The parameter *α* is calculated for thick-walled pipes (*D*/*e* < 25) anchored along their length [11]:

(4) $$\alpha =\left(1-\nu_{P}^{2}\right)\frac{D}{D+e}+\left(1+\nu_{P}\right)\frac{2e}{D}$$

In laminar flow, the friction factor is a function of the kinematic viscosity, flow velocity, and pipe inner radius (through the Reynolds number):

(5) fν,v,R=64Re=64νvD=32νvR

In turbulent flow, the friction factor is determined from the implicit Colebrook–White equation and is additionally a function of the average pipe wall roughness, *ε*:

(6) $$\frac{1}{\sqrt{f}}=-2\log\left(\frac{\varepsilon }{7.4R}+\frac{1.255\nu }{\mathrm{vR}\sqrt{f}}\right)$$

In this work, the most accurate Goudar–Sonad [16] approximation of the above implicit equation is used.

As there are no full analytical solutions to the presented water hammer equations (Equation (2)), and since ready-to-use computer applications [17] enabling transient flow simulations with acceptable accuracy are still lacking, this work applies the numerical method of characteristics. Using this method, the solution for an inner grid node is obtained:

(7) $$\left\{\begin{array}{c} p_{i,t+\Delta t}=\frac{1}{2}\left[\left(p_{i-1,t}+p_{i+1,t}\right)+\mathrm{c\rho}\left(v_{i-1,t}-v_{i+1,t}\right)+\frac{2c\Delta t}{R}\left(\tau_{i+1,t}-\tau_{i-1,t}\right)\right]\\ v_{i,t+\Delta t}=\frac{1}{2}\left[\left(v_{i-1,t}+v_{i+1,t}\right)+\frac{1}{\mathrm{c\rho}}\left(p_{i-1,t}-p_{i+1,t}\right)-\frac{2\Delta t}{\mathrm{\rho R}}\left(\tau_{i+1,t}+\tau_{i-1,t}\right)\right] \end{array}\right.$$

The wall shear stress *τ* is calculated from the corrected efficient convolution integral solution [18]:

(8) $$\tau_{\mathrm{ii},t}=\frac{\rho f_{\mathrm{ii},t}v_{\mathrm{ii},t}\left|v_{\mathrm{ii},t}\right|}{8}+\frac{2\rho \nu }{R}\sum_{j=1}^{3}\underset{y_{j,\mathrm{ii},t+∆t}}{\underbrace{\left[A_{j}y_{j,\mathrm{ii},t}+\eta B_{j}\left[v_{\mathrm{ii},t}-v_{\mathrm{ii},t-\Delta t}\right]+\left[1-\eta \right]C_{j}\left[v_{\mathrm{ii},t-\Delta t}-v_{\mathrm{ii},t-2\Delta t}\right]\right]}}$$

where index *ii* is *i* + 1 or *i* − 1 depending on which *τ* is calculated, and:

(9) $$A_{j}=e^{-n_{j}\Delta \hat{t}};\;B_{j}=\frac{m_{j}}{\Delta \hat{t}n_{j}}\left(1-A_{j}\right);\;C_{j}=A_{j}B_{j}$$

The coefficients *m_j_* and *n_j_* are calculated using the analytical solution presented in work [19].

## 3. Role of the Parameters

**Figure 1:**
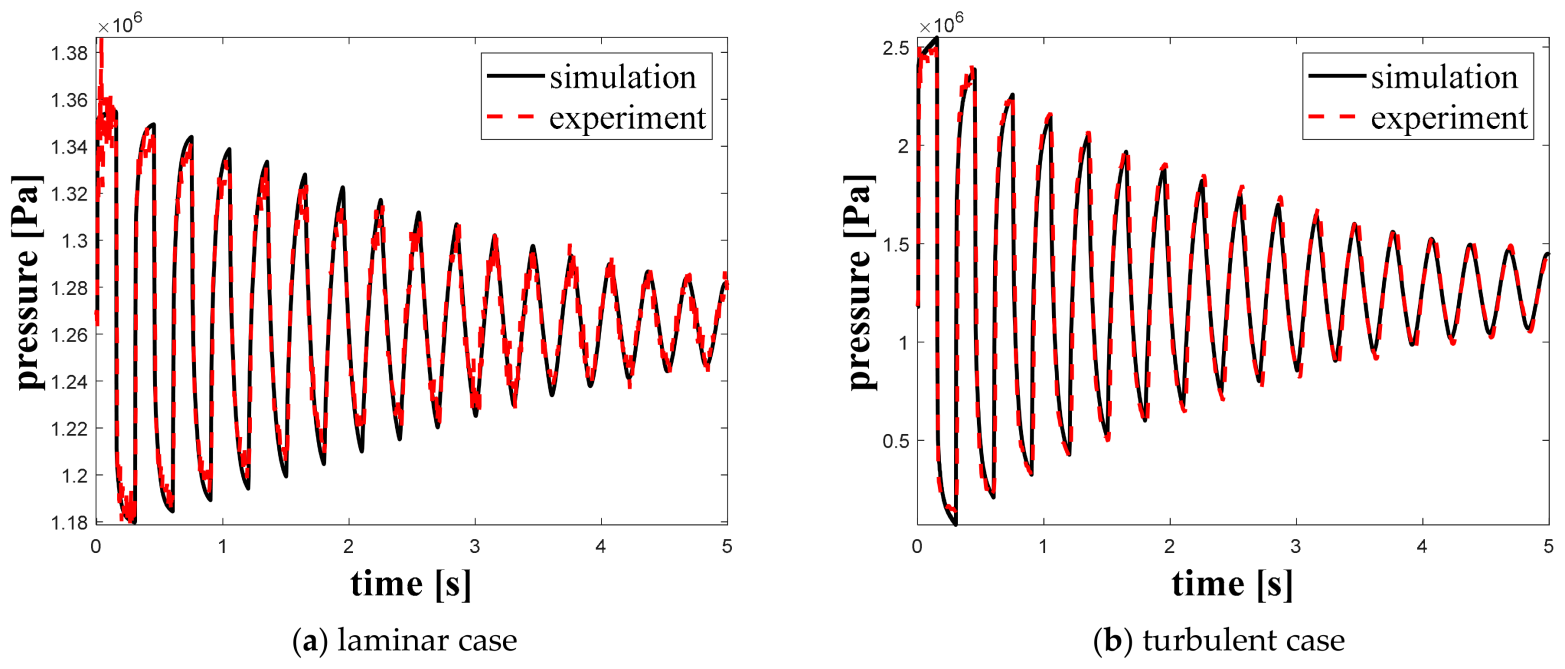
Fit of simulation results for initial values of tested parameters.

In this section, the influence of individual parameters appearing in the equations discussed in Section 2, which affect the simulated transient runs, is investigated. The initial values of the analyzed parameters (Table 1—second column) were adopted from experimental studies reported in the literature, carried out in Gdańsk at IMP PAN by Adamkowski and Lewandowski [20]. The experimental setup consisted of two reservoirs connected by a copper pipe of length *L* = 98.11 m. From the analysis of the recorded experimental dynamic pressure traces, the actual pressure wave propagation velocity in the tested system was determined as *c* = 1311.35 m/s. For this velocity *c*, with the other parameters kept constant (second column of Table 1) and with a numerical discretization of the pipe into *N* = 32 computational sections, qualitative agreement was obtained between the simulated and experimental pressure histories for both laminar flow (*v*_0_ = 0.066 m/s) and turbulent flow (*v*_0_ = 0.94 m/s), as shown in Figure 1. The quantitative assessment of simulated and experimental pressure histories can be performed using the pressure and timing error coefficients *E_p_* and *E_t_*, respectively, following the methodology of Urbanowicz et al. [18]. In that study, *E_p_* quantifies the mean relative error in selected pressure peaks, whereas *E_t_* quantifies the mean relative error in their occurrence times. The mesh-refinement analysis reported by Urbanowicz et al. [18] showed that the preferred discretization depends on the accuracy criterion: *N* = 52 yielded the lowest pressure error *E_p_*, whereas *N* = 202 yielded the lowest timing error *E_t_*. In the present study, *N* = 32 was retained to provide a consistent and computationally economical discretization across the extensive parametric simulations. Thus, this choice is intended to support comparative sensitivity analysis rather than to minimize the numerical error for every response quantity.

In simulation calculations, hydraulic system data are often adopted inaccurately. This results from the lack of mathematical formulas enabling the determination of the necessary parameters representing the flowing liquid (*ρ*, *K*, *ν*) and the pipe material (*E*_0_, *ν_P_*) as functions of temperature and pressure. In addition, one must remember that pipes are manufactured within certain dimensional tolerances; therefore, the actual wall thickness *e* and internal radius *R* may differ from the nominal values given in manufacturer catalogues. To clearly illustrate the influence of the individual input parameters on the modeled flow, large deviations from the initial values were assumed. Accordingly, for all parameters summarized in Table 1, possible deviations of ±10% were considered. The corresponding minimum and maximum values of the analyzed parameters were then calculated and summarized in the fourth and fifth columns of Table 1.

**Figure 2:**
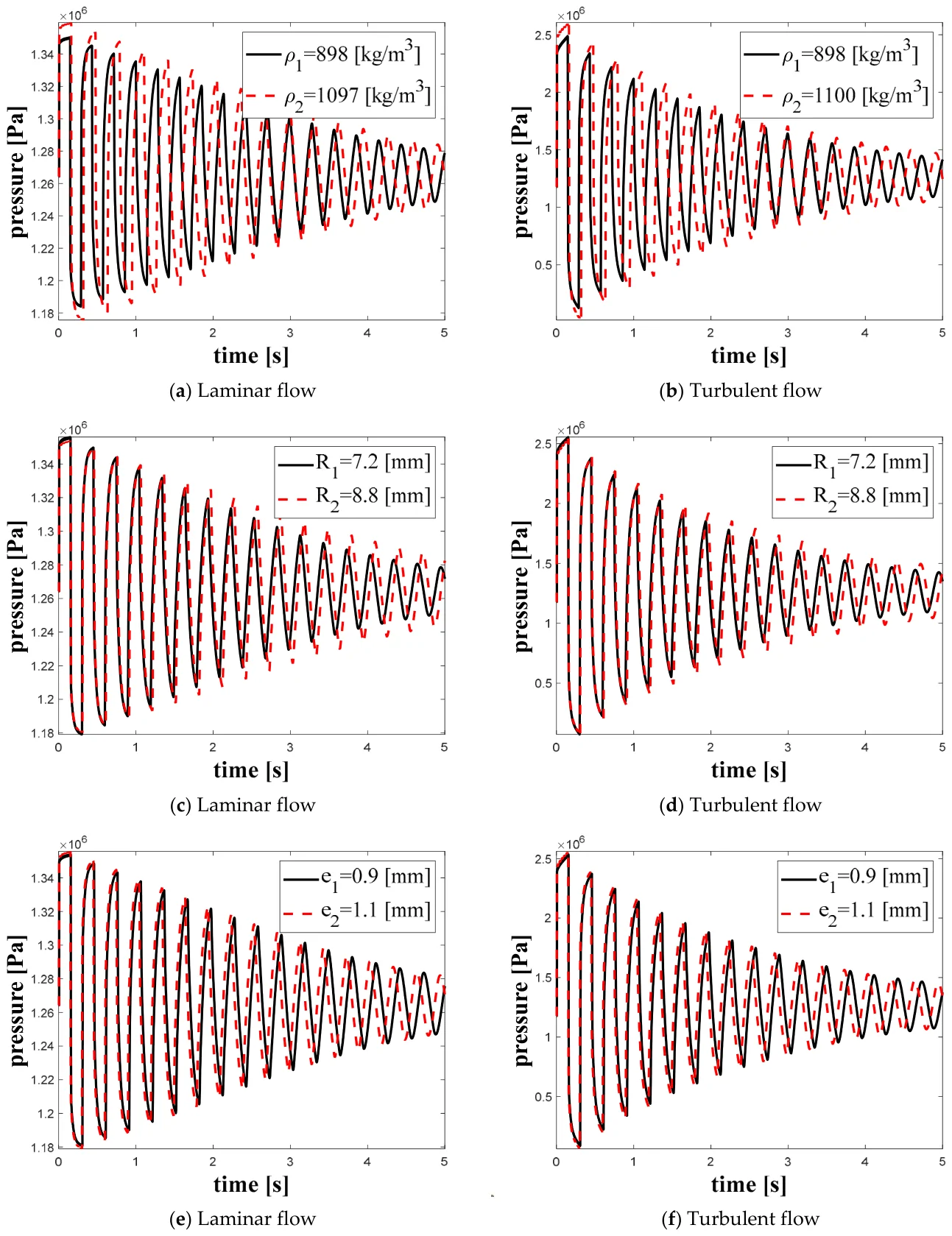
Liquid density *ρ*, inner pipe radius *R* and wall thickness *e* effects (left column: laminar flow results, right column: turbulent flow results).

**Figure 3:**
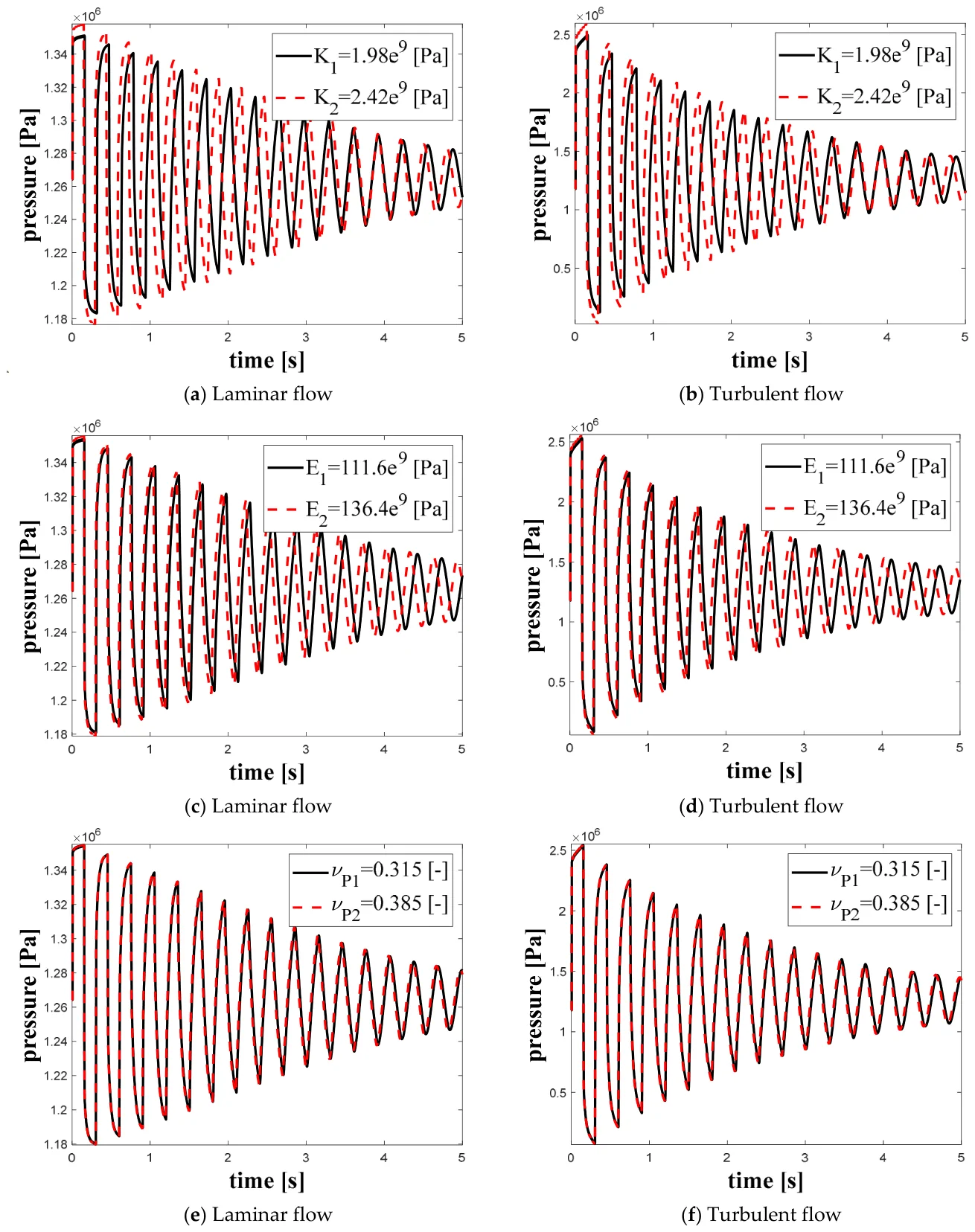
Liquid bulk modulus *K*, elastic modulus *E*_0_ and Poisson coefficient *ν_P_* effects (left column: laminar flow, right column: turbulent flow).

**Figure 4:**
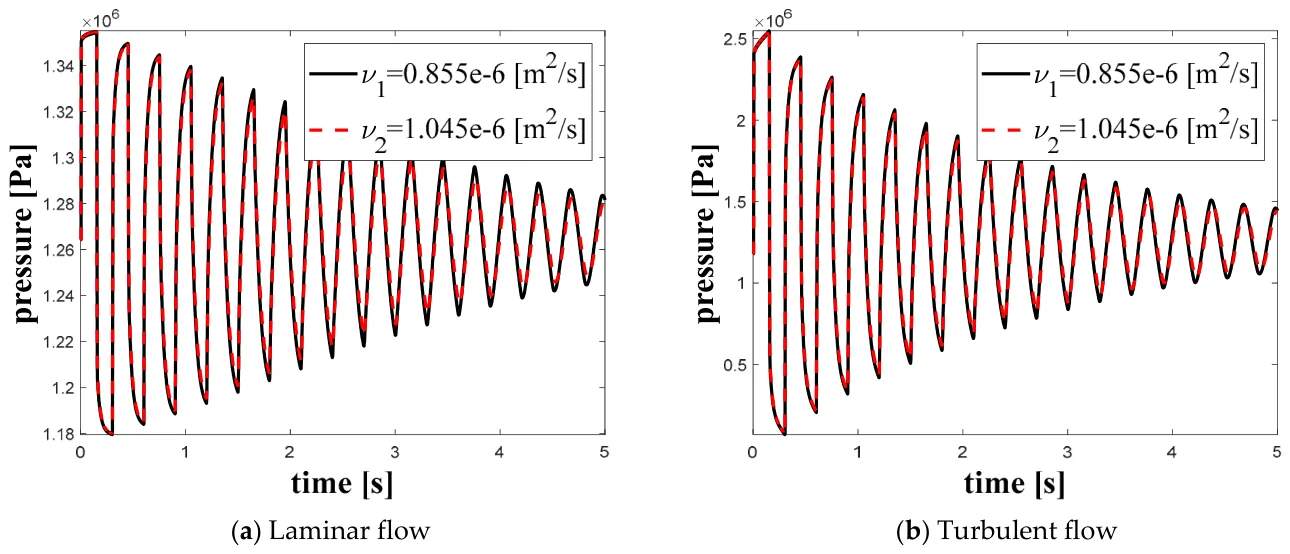
Liquid kinematic viscosity ***ν*** effect.

The results of the numerical simulations illustrating the influence of individual parameters are presented in Figure 2, Figure 3 and Figure 4. Among all the analyzed parameters, only the kinematic viscosity coefficient *ν* does not affect the value of the pressure wave propagation velocity. The detailed influence of the assumed parameters on the wave propagation velocity *c*, as well as the influence of selected parameters (*R*, *e*, and *ν_P_*) on the parameter describing the support condition of the pipe *α* [-], and on the enhanced *α*, expressed as a dimensionless coefficient denoted in this work by the symbol *Ξ*, are summarized in Table 2.

The effect of single-parameter changes is summarized briefly in Table 3, and other conclusions resulting from all performed research for single-parameter effects are:

(a) for the first amplitude *p*_1_ absolute pressure ↑: *ρ*↑, *R*↓, *e*↑, *ν_P_*↑, *K*↑, *E*_0_↑, *ν*—no effect!;
(b) for fifteen amplitude *p*_15_ absolute pressure ↑: *R*↑, *ν*↓;
(c) for fifteen amplitude arrival time *T*_15_ ↑: *ρ*↑, *R*↑, *e*↓, *ν_P_*↓, *K*↓, *E*_0_↓.

**Figure 5:**
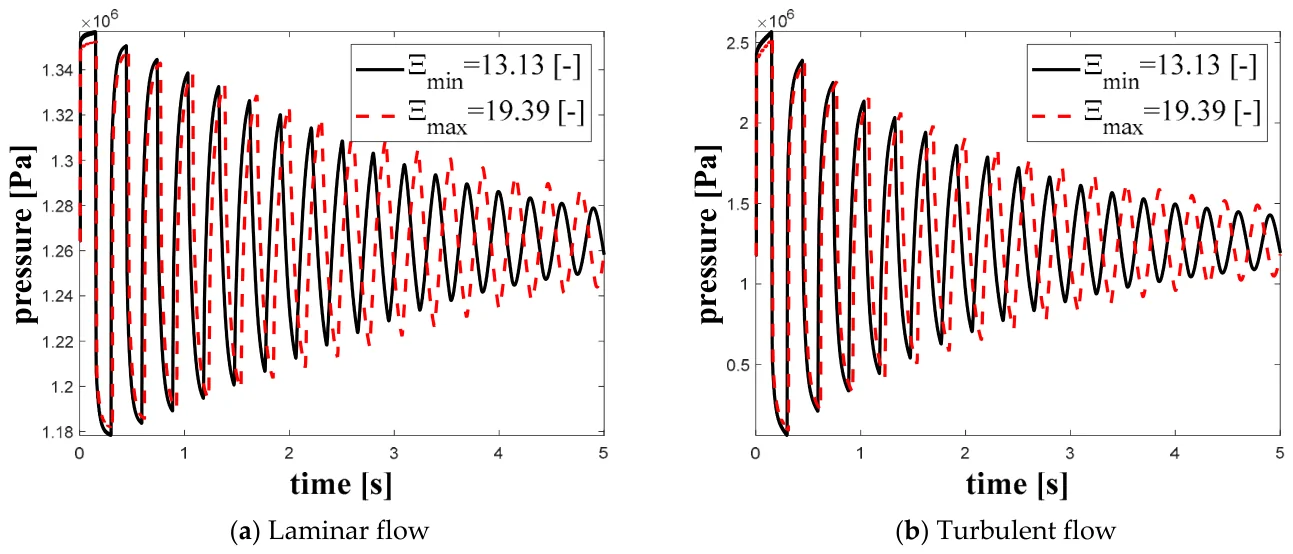

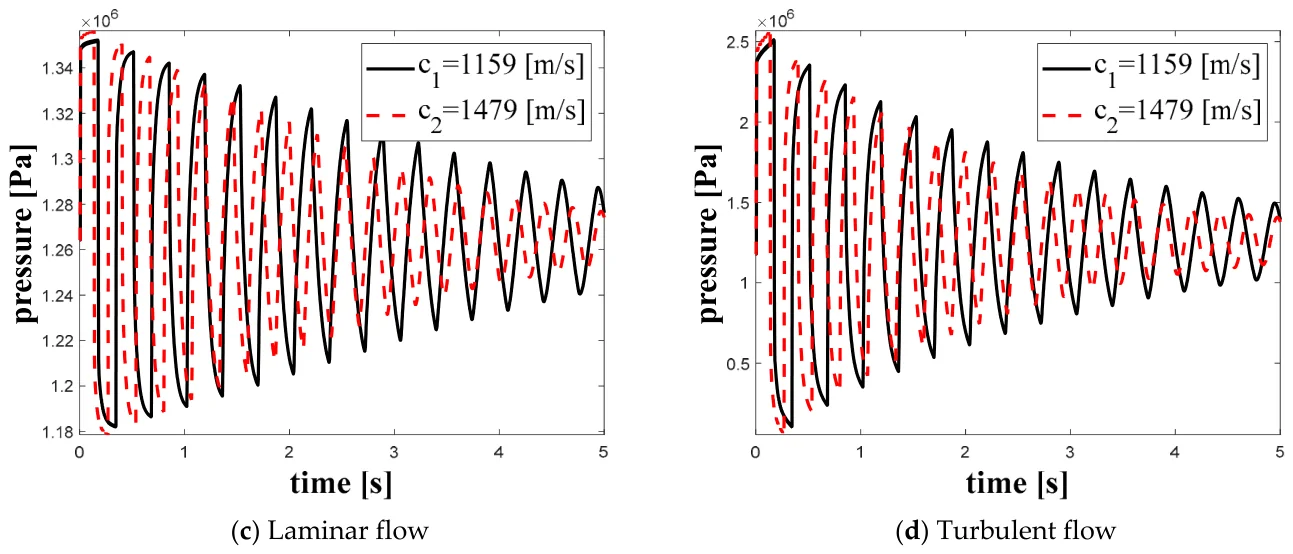
Multiparameter effects.

*Multiparameter* effect takes place when three parameter changes are assumed. When one assumes: *ν_P_*_2_ = 0.385, *R*_1_ = 0.0072 and *e*_2_ = 0.0011 then the dimensionless parameters takes values: *α* = 1.003, *Ξ* = 13.129 and pressure wave speed is *c* = 1337.4 m/s. Otherwise for: *ν_P_*_1_ = 0.315, *R*_2_ = 0.0088 and *e*_1_ = 0.0009, the resulting values are *α* = 0.991; *Ξ* = 19.388 and *c* = 1280.9 m/s. The differences in the final pressure runs are presented in Figure 5a,b. An *extreme case* takes place when assuming *ρ*_2_ = 1097, *R*_2_ = 0.0088, *e*_1_ = 0.0009, *K*_1_ = 1.98 × 10^9^, *ν_P_*_1_ = 0.315, *E*_1_ = 111.6 × 10^9^ in this case the calculated minimal pressure wave speed was: *c_min_* = 1159 m/s. When assuming *ρ*_1_ = 898, *R*_1_ = 0.0072, *e*_2_ = 0.0011, *K*_2_ = 2.42 × 10^9^, *ν_P_*_2_ = 0.385, *E*_2_ = 136.4 × 10^9^, the calculated maximal pressure wave speed was: *c_max_* = 1479 m/s. The difference in the pressure wave speed between the two extreme cases was 320 m/s. For *c_min_*, the number of pressure amplitudes that were simulated and analyzed 5 s after quick step closing of the valve was fifteen, and for *c_max_* the number of simulated amplitudes was nineteen (Figure 5c,d).

The obtained results showed that the temporal consistency of the modeled amplitudes depends strictly on the calculated pressure wave speed value. This velocity is influenced by six parameters assumed before the calculations. Two of them are related to fluid properties: *K* (bulk modulus) and *ρ* (fluid density). The remaining four are related to pipe properties in which the transient flow is analyzed: *E*_0_—Young’s modulus and *ν_P_*—Poisson’s ratio (material constants); *R*—pipe inner radius and *e*—pipe wall thickness (geometric properties). Analysis of the pressure values at the simulated fifteen amplitudes indicates that variations in the propagation velocity of the pressure wave are less significant than changes in the kinematic viscosity coefficient *ν* of the flowing fluid. This results from the fact that viscosity appears only in the final mathematical terms of the governing equations, which are responsible for modeling the hydraulic resistance of the flow.

## 4. Methodological Framework for Hydraulic System Analysis Under Water Hammer Conditions

Multiple-Valued Logic (MVL) goes beyond the rigid on/off world of binary by allowing signals to take more than two admissible values (three, four, or even a continuum) so information can be encoded more compactly, circuits can do more work per wire, and models can express graded states rather than hard thresholds. A mature theoretical and representational base underpins this idea: surveys by Miller & Thornton [21] map out MVL’s core concepts and the decision-diagram formalisms that make multi-valued functions tractable in software and hardware, including their quantum-aware extensions. At the same time, practical implementation has evolved along several lines. On the algorithmic side, Danesh & Rahman [22] show that machine-learning heuristics (iterative linear regressions plus pattern matching) can decompose multi-valued functions into hardware-friendly blocks, exemplified by a quaternary multiplier. On the reliability/logic side, Garribba et al. introduced MVL trees [23] that reason directly with multi-level states, grouping behaviors into Classes of Equivalent Behaviors and extracting prime implicants with tabular and Nelson-style procedures, an early demonstration that MVL can capture system reliability patterns more naturally than binary cut-set logic. Broad overviews (e.g., Gaudet’s guest editorial) highlight a recurring trade-off: higher radices promise better interconnect efficiency and area utilization but tighten noise margins and increase sensitivity to process variation, making device-level robustness a first-class concern [24]. This tension has been recognized since the classic “prospects” and tutorial papers by Smith, which framed MVL’s benefits (coding density, arithmetic compactness) alongside layout, power, and tooling realities [25,26].

Toolchains and devices have likewise diversified. Ordered multi-valued decision diagrams (OMDDs) enable formal verification of MVL networks, while multi-valued decision diagram MDD-based synthesis scales up logic construction without exploding into binary multiplexers [27,28,29]. At the architecture level, look-up table (LUT) concepts extend naturally to multi-valued field-programmable gate array FPGAs, reducing transistor count and enabling denser mappings [30,31,32,33], and even analog reconfigurable arrays have been used to realize continuous, fuzzy, and MVL behavior in silicon [34].

Although MVL and related decision-tree representations provide a powerful framework for modeling complex engineering systems, their practical application often requires an appropriate reduction in the parameter space and identification of dominant system variables [35].

The OFAT approach [35,36] is first employed to identify the dominant hydraulic parameters governing the transient response of the analyzed system. Subsequently, the obtained sensitivity information is utilized for the construction of interpretable decision structures and CART-based regression trees [37].

This combination enables both physical interpretation of the governing hydraulic mechanisms and data-driven representation of the relationships between system parameters and transient responses.

The present study adopts an integrated methodological framework combining OFAT sensitivity analysis, rule-based decision structures, and CART regression models. The OFAT method is first used to quantify the individual influence of selected hydraulic parameters on the transient response and to identify the dominant variables under laminar and turbulent flow conditions.

The obtained sensitivity results are subsequently used to develop interpretable rule-based decision structures, which organize the dominant parameter–response relationships into qualitative classes. CART regression models are then employed to represent the observed relationships through data-driven hierarchical splits and to assess how well the resulting models reproduce the investigated OFAT responses.

### 4.1. OFAT Sensitivity Analysis

OFAT method is used to evaluate the influence of individual parameters on the system response. The method is based on independent perturbation of each parameter while keeping the remaining parameters fixed.

The analyzed system is described by a general functional relationship:

*Y* = *f*(*x*_1_, *x*_2_, …, *x_n_*) (10)

where *x_i_* are the input parameters and *Y* is the system response.

(11) $$\left\{\begin{array}{c} X_{0}=\left(x_{1,0},x_{2,0},\ldots ,x_{n,0}\right)\\ Y_{0}=f\left(X_{0}\right) \end{array}\right.$$

In the OFAT approach, a single parameter *x_i_* is perturbed:

*x_i_*(*δ*) = *x_i_*_,0_(1 + *δ*) (12)

*x_j_* = *x_j_*_,0_,   *j* ≠ *i* (13)

The corresponding system response is given by:

*Y_i_*(*δ*) = *f*(*x*_1,0_,…,*x_i_*(*δ*),…,*x_n_*_,0_) (14)

The influence of the parameter is quantified using the absolute deviation from the baseline case:

Δ*Y_i_*(*δ*) = *Y_i_*(*δ*) − *Y*_0_ (15)

and the relative sensitivity:

(16) $$S_{i}\left(\delta \right)=\frac{Y_{i}\left(\delta \right)-Y_{0}}{Y_{0}}$$

In percentage form:

(17) $$S_{i}^{\left(\%\right)}\left(\delta \right)=\frac{Y_{i}\left(\delta \right)-Y_{0}}{Y_{0}}\cdot 100\%$$

To evaluate the overall influence of parameter *x_i_* the response range is defined as:

(18) $$R_{i}=\underset{\delta }{\max}Y_{i}\left(\delta \right)-\underset{\delta }{\min}Y_{i}\left(\delta \right)$$

A normalized impact coefficient is introduced as:

*I_i_* = (*R_i_*/*Y*_0_)∙100% (19)

*I_i_*—normalized impact coefficient for the i-th parameter,

*R_i_*—response range defined by Equation (18),

*Y*_0_—baseline value of the analyzed response.

A local sensitivity coefficient can be approximated using a finite-difference scheme.

(20) $$\frac{\partial Y}{\partial x_{i}}\approx \frac{Y\left(x_{i}+\Delta x_{i}\right)-Y\left(x_{i}-\Delta x_{i}\right)}{2\Delta x_{i}}$$

The normalized form of this coefficient is given by:

(21) $$S_{i}^{*}=\frac{\frac{Y\left(x_{i}+\Delta x_{i}\right)-Y\left(x_{i}-\Delta x_{i}\right)}{Y_{0}}}{\frac{\left(x_{i}+\Delta x_{i}\right)-\left(x_{i}-\Delta x_{i}\right)}{x_{i,0}}}$$

For qualitative interpretation, the parameter influence is classified as:

(22) $$D_{i}\left(\delta \right)=\left\{\begin{array}{cc} ↑, & \Delta Y_{i}\left(\delta \right)>0\\ ↓, & \Delta Y_{i}\left(\delta \right)<0\\ \approx , & \Delta Y_{i}\left(\delta \right)\approx 0 \end{array}\right.$$

The OFAT approach evaluates the influence of each hydraulic parameter separately, while all remaining parameters are maintained at their baseline values. Therefore, the adopted experimental design does not provide information on interaction effects between simultaneously varied parameters. The resulting sensitivity coefficients describe individual parameter effects within the investigated perturbation ranges.

### 4.2. Analysis of Parameter Influence

The transient hydraulic response is described by the mapping:

**Y** = *f*(**X**) (23)

where the input vector is defined as:

**X** = [*ρ*, *R*, *e*, *ν_P_*, *ν*, *K*, *E*] (24)

and the output vector is given by:

**Y** = [*T*_15_, *p*_15_] (25)

The baseline (reference) state is defined as:

(26) $$\left\{\begin{array}{c} X_{0}=\left[\rho_{0},R_{0},e_{0},\nu_{P,0},\nu_{0},K_{0},E_{0}\right]\\ Y_{0}=f\left(X_{0}\right) \end{array}\right.$$

For each parameter, four perturbation levels were considered independently −10%, −5%, +5%, +10%. In each case, only one parameter was varied, while the remaining parameters were kept at their baseline values, denoted by the subscript *b:*

(27) $$\rho_{b}=997.65\;\frac{\mathrm{kg}}{m^{3}},\;R_{b}=0.008\;m,\;e_{b}=0.001\;m,\;\nu_{P,b}=0.35,\;\nu_{b}=0.95\times 10^{-6}\;m^{2}/s,\;K_{b}=2.2\times 10^{9}\;\mathrm{Pa},E_{b}=124\times 10^{9}\;\mathrm{Pa}.$$

For each perturbation, the resulting system response was evaluated in terms of:

*T*_15_ [s]   and   *p*_15_ [Pa] (28)

The analysis was performed separately for laminar and turbulent flow regimes, with the following baseline values:

- Laminar flow—*T*_15_ = 4.370 s and *p*_15_ = 1.287 × 10^6^ Pa;
- Turbulent flow—*T*_15_ = 4.374 s and *p*_15_ = 1.495 × 10^6^ Pa.

The parameter perturbation ranges (±5% and ±10%) were selected as prescribed sensitivity-analysis scenarios to evaluate the response of the model to small and moderate changes in the baseline parameters. Applying the same relative perturbations to all parameters provides a consistent basis for comparing their influence on the calculated transient response. However, these ranges should not be interpreted as universal estimates of manufacturing tolerances or uncertainties in material and liquid properties. Actual dimensional tolerances depend on the pipe material, manufacturing process, nominal dimensions, and applicable product standards. Similarly, the uncertainty and variability of liquid properties, including density, bulk modulus, and kinematic viscosity, depend on liquid composition, temperature, pressure, and the methods used to determine these properties. The selected perturbations therefore represent a controlled comparative sensitivity study rather than a probabilistic uncertainty analysis or a direct representation of tolerances for a particular piping system. Establishing application-specific uncertainty ranges would require parameter-specific information from material certificates, manufacturing specifications, property measurements, or relevant technical data.

The results of the OFAT sensitivity analysis for individual parameters are summarized in Table A1 (found in Appendix A). The table presents the response of the system in terms of *T*_15_ and *p*_15_ for different levels of parameter perturbation, while all remaining parameters were kept at their baseline values. Table A1 shows that the influence of individual parameters varies in magnitude and depends on the flow regime. Some parameters have a noticeable impact on *T*_15_, while others affect the response only marginally. The sensitivity of *p*_15_ is generally lower in laminar flow and more pronounced in turbulent conditions. These results indicate that only selected parameters play a dominant role in shaping the transient response.

The response range defined in Table 4 represents the total variation in the output caused by perturbations of a given parameter.

(29) $$I_{k,i}=\frac{R_{k,i}}{Y_{k,0}}\cdot 100\%$$

For example, for *ρ*:

(30) $$R_{T_{15},\rho }=T_{15}^{\max}-T_{15}^{\min}$$

(31) $$R_{T_{15},\rho }=4.585-4.143=0.442$$

The calculated values of the response range and the corresponding normalized impact coefficients for all analyzed parameters are presented in Table 4 for laminar and turbulent flow regimes, respectively.

The response ranges and the corresponding normalized impact coefficients presented in Table 4 provide the basis for evaluating the influence of individual hydraulic parameters on the transient hydraulic response. Since the OFAT sensitivity analysis was performed independently for laminar and turbulent flow conditions, the obtained parameter rankings are discussed separately for each flow regime. This approach enables identification of both common and flow-specific mechanisms governing the temporal response *T*_15_ and the pressure response *p*_15_.

### 4.3. Sensitivity of Transient Temporal and Pressure Responses

**Figure 6:**
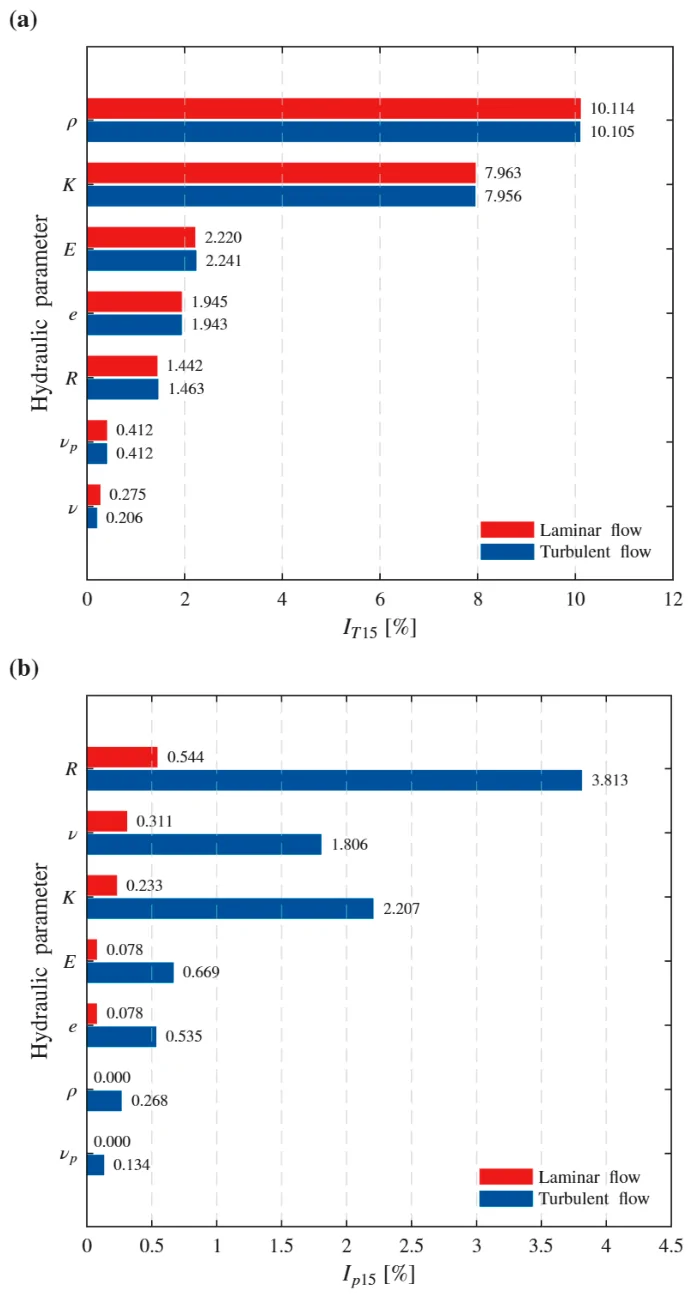
Normalized impact coefficients quantifying the relative influence of the investigated hydraulic parameters on (**a**) the temporal response *T*_15_ and (**b**) the pressure response *p*_15_ under laminar and turbulent flow conditions. Red and blue bars denote laminar and turbulent flow, respectively.

The normalized impact coefficients obtained from the OFAT analysis (Table 4) reveal distinct sensitivity patterns for the temporal response *T*_15_ and pressure response *p*_15_ under laminar and turbulent flow conditions (Figure 6). The corresponding parameter hierarchies, obtained by ranking the normalized impact coefficients in descending order, are

(32) $$\left\{\begin{array}{c} T_{15}^{\mathrm{lam}}:\;\rho >K>E>e>R>\nu_{p}>\nu ,\\ T_{15}^{\mathrm{turb}}:\;\rho >K>E>e>R>\nu_{p}>\nu ,\\ p_{15}^{\mathrm{lam}}:\;R>\nu >K>E\approx e>\rho \approx \nu_{p},\\ p_{15}^{\mathrm{turb}}:R>K>\nu >E>e>\rho >\nu_{p}. \end{array}\right.$$

For *T*_15_, the ranking is essentially unchanged between the two flow regimes, with fluid density *ρ* exhibiting the greatest influence, followed by the bulk modulus *K* and Young’s modulus *E*. The wall thickness *e* and pipe radius *R* have substantially lower effects, whereas the Poisson’s ratio *ν_p_* and kinematic viscosity *ν* contribute only marginally. The close agreement between the two rankings indicates that the dominant factors governing *T*_15_ are largely insensitive to the flow regime within the investigated parameter ranges (Figure 6a).

In contrast, the sensitivity of *p*_15_ is strongly dependent on the flow regime. Under laminar conditions, the pipe radius *R* is the dominant parameter, followed by the kinematic viscosity *ν* and bulk modulus *K*, while the remaining parameters have comparatively limited effects. Under turbulent conditions, *R* remains the most influential parameter, but the effects of *K* and *ν* increase markedly (Figure 6b). Specifically, the normalized impact coefficient of *K* increases from 0.233 under laminar flow to 2.207 under turbulent flow, while that of *ν* increases from 0.311 to 1.806. This pronounced increase indicates a stronger sensitivity of the pressure response to fluid compressibility and viscous effects under turbulent transient conditions.

The negligible normalized impact coefficients obtained for fluid density (*ρ*) and Poisson’s ratio (*ν_p_*) in the laminar pressure response require additional physical interpretation. Here, the values are obtained by rounding the pressure values to three decimal places in MPa, which may mask small pressure variations and yield zero normalized impact coefficients. According to the governing equations, fluid density affects the relationship between pressure and flow velocity as well as pressure-wave propagation, whereas Poisson’s ratio contributes to pipe-wall compliance and consequently influences wave celerity. Therefore, the reported zero coefficients should not be interpreted as indicating the absence of a physical influence of these parameters on water-hammer phenomena.

It is important to note that the present analysis considers the pressure associated with the fifteenth pressure maximum (*p*_15_), rather than the initial pressure rise or the maximum pressure reached during the entire transient. The influence of individual parameters on this specific pressure indicator may therefore differ from their influence on other characteristics of the hydraulic response. This distinction is particularly evident for fluid density: while the reported *p*_15_ values remain unchanged under the investigated laminar-flow perturbations, the corresponding temporal response *T*_15_ varies from 4.143 to 4.585 s. Thus, density substantially affects the timing of pressure-wave propagation, even though no variation is observed in the reported *p*_15_ values. Similarly, Poisson’s ratio influences the elastic response of the pipe wall, but its effect on the selected pressure indicator is not resolved in the reported laminar-flow results. These findings demonstrate that the normalized impact coefficients describe the sensitivity of specific response measures within the investigated OFAT parameter ranges and should not be generalized to the overall water-hammer phenomenon.

The observed sensitivity patterns can be explained by the physical mechanisms governing pressure-wave propagation and unsteady friction. The temporal response, represented by *T*_15_, is primarily associated with pressure-wave travel time and therefore depends on wave celerity, which is governed by the compressibility of the liquid and the elastic compliance of the pipe wall. The liquid density *ρ* contributes to fluid inertia, whereas the bulk modulus *K* characterizes the resistance of the liquid to compression. For a given pipe configuration, an increase in *K* generally increases the pressure-wave celerity, while an increase in *ρ*, with the other parameters held constant, generally decreases it. Young’s modulus *E* determines the stiffness of the pipe wall: a higher value reduces elastic wall deformation and generally increases wave celerity. The aforementioned shifts in wave celerity are also supported by Equation (3). These changes modify the wave-travel time and therefore the timing of successive pressure maxima. This interpretation is consistent with the similar parameter rankings obtained for *T*_15_ under laminar and turbulent flow conditions, indicating that the temporal response is governed predominantly by liquid compressibility and pipe-wall elasticity within the investigated parameter ranges.

The pressure response *p*_15_ exhibits a different sensitivity pattern because it reflects not only wave propagation but also the evolution of the transient pressure field and the cumulative effects of friction. Pipe radius *R* was the dominant parameter for *p*_15_ under both flow regimes. This influence is associated with the role of pipe geometry in determining wall compliance, cross-sectional area, and the relationship between pressure and fluid velocity. Changes in radius can therefore affect both wave celerity and the pressure response predicted by the governing equations. The liquid bulk modulus also influences pressure-wave propagation through liquid compressibility and, for a prescribed velocity change, affects the pressure rise through the Joukowsky relation, ∆*p* = *cρ*∆*v*, under the usual assumptions of this relation. However, the pressure response analyzed here is *p*_15_, the fifteenth pressure maximum, rather than the initial pressure rise; consequently, its sensitivity also reflects the evolution of the transient over successive wave cycles.

Kinematic viscosity *ν* influences the transient response through frictional resistance and the development of the velocity profile. In the adopted convolution-based formulation, unsteady friction represents the history-dependent contribution of viscous effects to the pressure and velocity fields. These effects influence the dissipation of transient energy and the evolution of successive pressure maxima. The greater sensitivity of *p*_15_ to viscosity under turbulent conditions is consistent with the different frictional behavior of the two flow regimes. The increased sensitivity to *K* under turbulent conditions also indicates that the pressure response at the selected wave cycle depends on the coupled effects of compressibility and friction. These findings should be interpreted within the parameter ranges and the specific response measure investigated, rather than as universal rankings for all water-hammer events.

Overall, the OFAT analysis demonstrates that the sensitivity of the transient hydraulic response depends on the selected response measure. The temporal response *T*_15_ is most sensitive to liquid density and bulk modulus, whereas the pressure response *p*_15_ is dominated by pipe radius and exhibits greater sensitivity to viscosity and bulk modulus under turbulent conditions. These findings provide a quantitative basis for selecting influential variables for the subsequent development of interpretable decision-tree models and IF–THEN engineering rules.

## 5. Development of Decision Structures for Hydraulic System Analysis

### 5.1. Theoretical Background of Decision Structures

Decision structures constitute a mathematical and logical framework used to describe relationships between input parameters and the corresponding system response. In hydraulic systems subjected to water hammer, decision structures enable identification of dominant parameters, formulation of interpretable rules, and classification of system behavior under different operating conditions.

In general form, the analyzed hydraulic system may be represented as *Y* = *f*(*X*), where *X* = [*x*_1_,*x*_2_,…,*x_n_*] is the vector of input parameters, while *Y* = [*y*_1_,*y*_2_,…,*y_m_*] represents the vector of response variables. In the present study, the input vector is defined as *X* = [*ρ*, *R*, *e*, *ν_P_*, *ν*, *K*, *E*] and the response vector is given by *Y* = [*T*_15_, *p*_15_].

The purpose of the decision structure is to transform the continuous relationship *Y* = *f*(*X*) into a set of interpretable logical rules describing the system behavior. A general decision rule may be expressed in the form:

IF *C*(*X*) THEN *Y* ∈ Ω (33)

The decision structures were constructed using the results obtained from the OFAT sensitivity analysis. Each record corresponds to a single perturbation experiment in which one parameter was modified while the remaining parameters were maintained at their baseline values. The obtained dataset constitutes the basis for extraction of decision rules and construction of interpretable decision trees.

For each parameter, four perturbation levels were considered δ = [−10%, −5%, +5%, +10%]. The dataset includes results obtained for both laminar and turbulent flow regimes. The structure of the dataset used for construction of decision structures is presented in Table 5.

For the pressure response variable *p*_15_, the following classification was introduced:

(34) $$p_{15}\in \left\{\begin{array}{cc} \mathrm{Low}, & p_{15}<p_{L}\\ \mathrm{Medium}, & p_{L}\leq p_{15}<p_{H}\\ \mathrm{High}, & p_{15}\geq p_{H} \end{array}\right.$$

Similarly, for the temporal response variable:

(35) $$T_{15}\in \left\{\begin{array}{cc} \mathrm{Short}, & T_{15}<T_{L}\\ \mathrm{Medium}, & T_{L}\leq T_{15}<T_{H}\\ \mathrm{Long}, & T_{15}\geq T_{H} \end{array}\right.$$

The threshold values *T_L_*, *T_H_*, *p_L_*, and *p_H_* were determined separately for the laminar and turbulent datasets based on the distribution of the corresponding OFAT response values. For each response case, the lower and upper thresholds were calculated from the observed response range, dividing it into three qualitative intervals corresponding to Low/Short, Medium, and High/Long response classes. Consequently, the classification thresholds were determined independently for $T_{15}^{\mathrm{lam}}$, $T_{15}^{\mathrm{turb}}$, $p_{15}^{\mathrm{lam}}$ and $p_{15}^{\mathrm{turb}}$, thereby preserving the differences between the two flow regimes. The numerical threshold values used for each case are reported together with the corresponding decision-structure analysis.

The transformation of continuous response variables into qualitative classes simplifies the construction of decision structures and improves interpretability of the resulting rules. Based on the separate OFAT ranking analyses performed for laminar and turbulent flow conditions, the dominant hydraulic parameters were selected for further decision structure development. For the temporal response *T*_15_, both flow regimes identified the same dominant parameters, namely the fluid density *ρ*, bulk modulus *K*, and Young’s modulus *E*. For the pressure response *p*_15_, slight differences were observed between laminar and turbulent flow conditions. Nevertheless, considering both analyses, the pipe radius *R*, bulk modulus *K*, and kinematic viscosity *ν* were selected as the most representative parameters for constructing the decision structures. The reduction in the parameter space decreases the complexity of the decision model while preserving the dominant physical mechanisms governing the transient hydraulic response.

Accordingly, the following parameters were selected for the subsequent construction of decision structures:

- for the temporal response—*T*_15_: *ρ*,*K*,*E*;
- for the pressure response—*p*_15_: *R*,*K*,*ν*.

The reduction in the parameter space decreases the complexity of the decision structure and enables construction of more transparent and interpretable hydraulic decision models.

### 5.2. Construction of Rule-Based Decision Structures

Since the dataset was generated using one-factor-at-a-time perturbations, the constructed decision structures represent individual parameter–response relationships rather than multiparameter interactions. Although MVL-based representations can theoretically accommodate more complex relationships, their interpretation in the present study is restricted by the underlying experimental design. Consequently, the resulting rules and thresholds should be understood as interpretable descriptions of the observed OFAT responses and should not be interpreted as evidence of multiparameter interaction effects.

To preserve the distinction between the two analyzed flow regimes, the decision structures were developed separately for laminar and turbulent flow conditions. Consequently, four independent response cases were considered:

$$T_{15}^{\mathrm{lam}},\;T_{15}^{\mathrm{turb}},\;p_{15}^{\mathrm{lam}},\;p_{15}^{\mathrm{turb}}.$$

**Figure A1:**
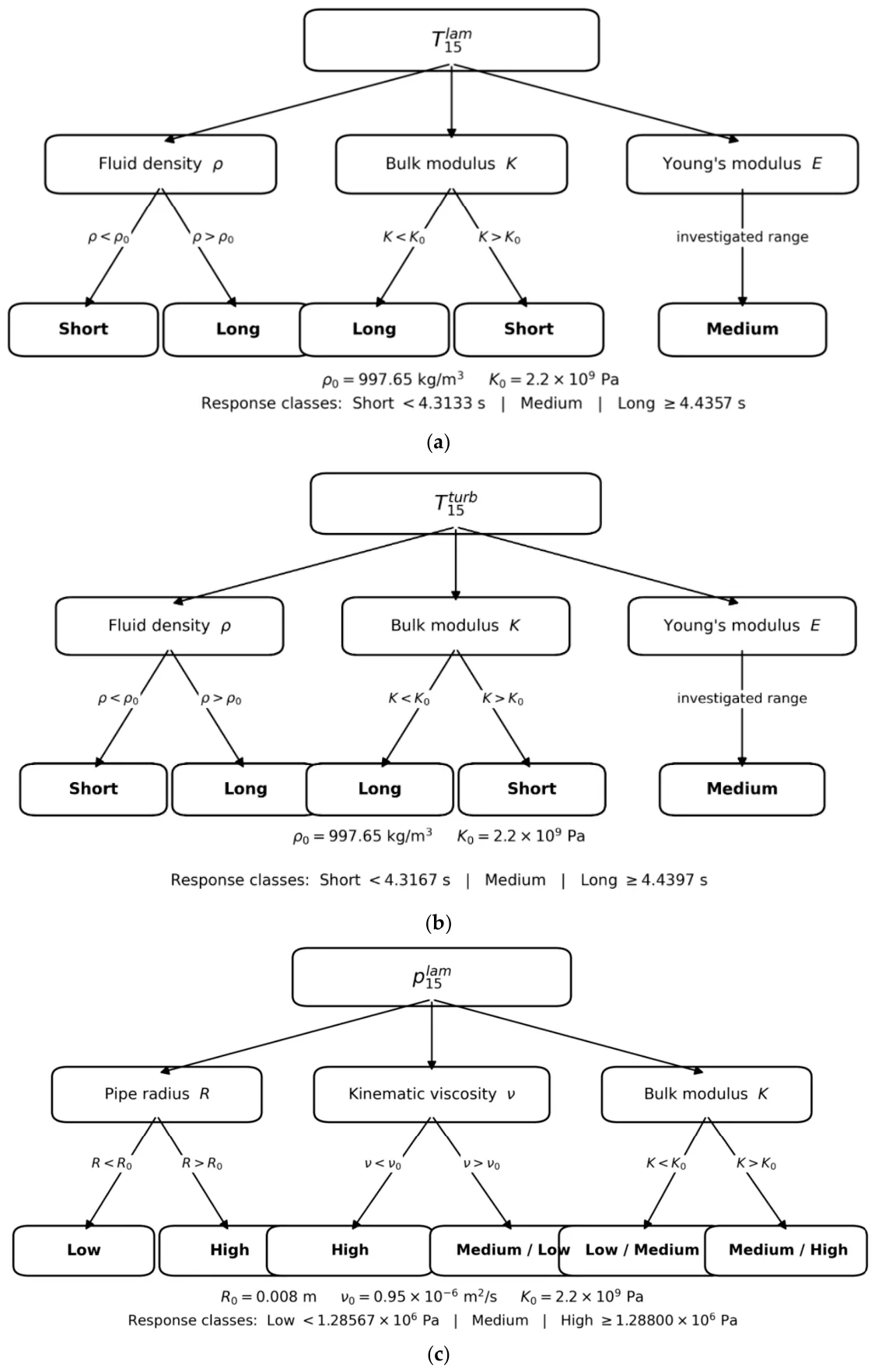

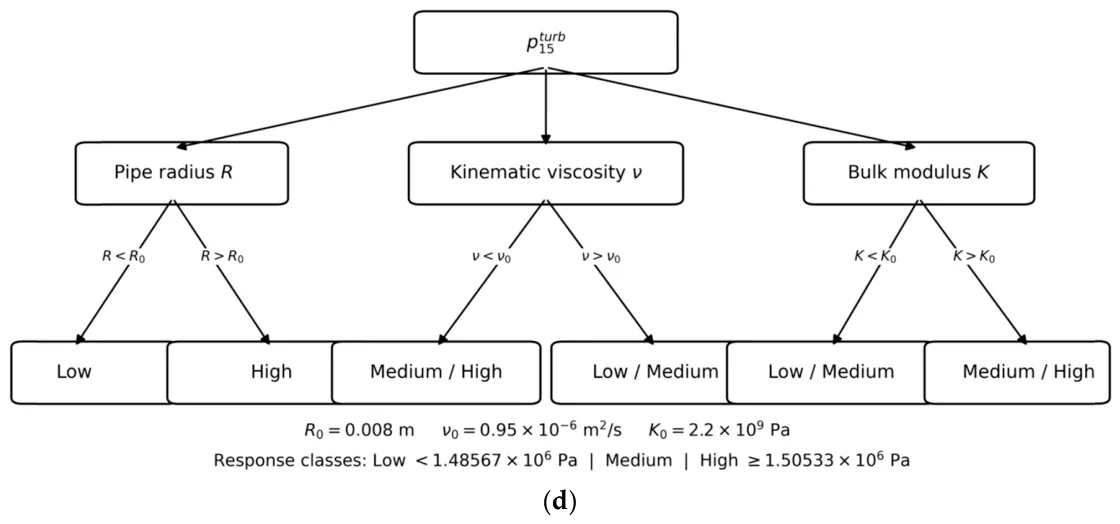
Rule-based decision structure for the temporal response: (**a**) *T*_15_ under laminar flow conditions; (**b**) *T*_15_ under turbulent flow conditions; (**c**) *p*_15_ under laminar flow conditions; (**d**) *p*_15_ under turbulent flow conditions.

For the temporal response, the qualitative classes **Short**, **Medium**, and **Long** were defined separately for the laminar and turbulent datasets with respect to the corresponding baseline values. Similarly, the pressure-response classes **Low**, **Medium**, and **High** were determined independently for each flow regime. This separation ensures that differences between laminar and turbulent transient behavior are preserved during the construction and interpretation of the decision structures. The simplified decision trees were therefore developed independently for each of the four response cases. Based on the dominant parameter relationships identified in the OFAT analysis, four separate rule-based decision trees were constructed. The resulting structures represent the temporal and pressure responses independently for laminar and turbulent flow conditions, as shown in Appendix A Figure A1a–d.

#### 5.2.1. Temporal Response *T*_15_ Under Laminar and Turbulent Flow Conditions

For both laminar and turbulent flow conditions, the rule-based decision structures for the temporal response *T*_15_ were constructed using the three dominant parameters identified by the OFAT analysis: fluid density *ρ*, bulk modulus *K*, and Young’s modulus *E*. The response values were divided into three qualitative classes, Short, Medium, and Long, using distribution-based thresholds determined independently for each flow regime. For laminar flow, the lower and upper thresholds were *T_L_*_, lam_ = 4.3133 s and *T_H_*_,lam_ = 4.4357 s, respectively. For turbulent flow, the corresponding thresholds were *T_L_*_,turb_ = 4.3167 s and *T_H_*_,turb_ = 4.4397 s. Thus, values below the lower threshold were classified as Short, values between the lower and upper thresholds as Medium, and values equal to or above the upper threshold as Long.

Table 6 presents the resulting *T*_15_ values and response classifications for the investigated perturbations under both flow conditions. The numerical values are highly similar between the laminar and turbulent cases, with only minor increases in *T*_15_ under turbulent flow. Importantly, the response classification remains unchanged for all investigated parameter variations.

The classification reveals consistent directional effects of fluid density *ρ* and bulk modulus *K* under both flow conditions. A decrease in fluid density relative to the baseline value results in a Short response, whereas an increase in *ρ* produces a Long response. The opposite tendency is observed for the bulk modulus *K*: lower values correspond to the Long class, while higher values result in a Short response. In contrast, variations in Young’s modulus *E* remain within the Medium class throughout the investigated perturbation range. Consequently, *ρ* and *K* provide a clear separation between the Short and Long temporal-response classes, whereas the influence of *E* is insufficient to shift the response beyond the Medium class.

The consistency of these trends across laminar and turbulent conditions indicates that the qualitative dependence of *T*_15_ on the dominant parameters is largely insensitive to the flow regime within the investigated range. The corresponding rule-based decision structures are presented in Figure A1a for laminar flow and Figure A1b for turbulent flow.

#### 5.2.2. Pressure Response *p*_15_ Under Laminar and Turbulent Flow Conditions

For both laminar and turbulent flow conditions, the rule-based decision structures for the pressure response *p*_15_ were constructed using the three dominant parameters identified by the sensitivity ranking: pipe radius *R*, kinematic viscosity *ν*, and bulk modulus *K*. The response values were divided into three qualitative classes, Low, Medium, and High, using distribution-based thresholds determined independently for each flow regime. For laminar flow, the lower and upper thresholds were *p_L_*_,lam_ = 1.28567 × 10^6^ Pa and *p_H_*_,lam_ = 1.28800 × 10^6^ Pa, respectively. For turbulent flow, the corresponding thresholds were *p_L_*_,turb_ = 1.48567 × 10^6^ Pa and *p_H_*_,turb_ = 1.50533 × 10^6^ Pa. Accordingly, values below the lower threshold were classified as Low, values between the lower and upper thresholds as Medium, and values equal to or above the upper threshold as High. The resulting classifications and *p*_15_ values for both flow regimes are presented in Table 7.

The classification reveals consistent directional relationships between the dominant parameters and the pressure response in both flow regimes. Pipe radius *R* provides the clearest separation between the response classes: a decrease in *R* relative to its baseline value results in a Low pressure response, whereas an increase in *R* produces a High response. Kinematic viscosity *ν* exhibits the opposite tendency, with lower values associated with higher *p*_15_, while increasing *ν* shifts the response toward the Medium and Low classes. The bulk modulus *K* shows a more gradual positive influence, with increasing *K* shifting the response from Low through Medium toward High.

Although the qualitative parameter–response relationships are consistent between laminar and turbulent conditions, the magnitude of the pressure response differs substantially between the two regimes. Under laminar flow, the investigated *p*_15_ values are concentrated within a relatively narrow range of approximately 1.283 × 10^6^ to 1.290 × 10^6^ Pa. In contrast, the turbulent-flow results span a considerably wider range, from approximately 1.468 × 10^6^ to 1.525 × 10^6^ Pa. This substantially wider response range is consistent with the greater pressure sensitivity under turbulent flow conditions identified previously by the OFAT analysis.

Thus, the rule-based structures based on *R*, *ν*, and *K* preserve the same main directional dependencies in both flow regimes, while the independently determined thresholds capture the regime-specific response ranges. The corresponding rule-based decision structures are presented in Figure A1c for laminar flow and Figure A1d for turbulent flow.

Overall, the four rule-based decision structures developed for *T*_15_ and *p*_15_ demonstrate that separating the laminar and turbulent flow regimes preserves the regime-specific characteristics of both temporal and pressure responses. For *T*_15_, the dominant relationships associated with *ρ*, *K*, and *E* remain similar between the two regimes, although the response-class thresholds differ slightly. For *p*_15_, the structures based on *R*, *ν*, and *K* likewise exhibit consistent directional relationships, but with substantially different response ranges and greater pressure sensitivity under turbulent flow. These rule-based structures therefore provide an interpretable representation of the principal parameter–response relationships identified by the OFAT analysis. However, because they primarily describe qualitative response classes and directional dependencies, a quantitative regression-based approach using CART models was additionally applied in the next stage.

### 5.3. CART-Based Decision Tree Modeling

To complement the qualitative rule-based structures presented in Section 5.2, CART modeling was applied to obtain quantitative and interpretable representations of the hydraulic system response. Since both *T*_15_ and *p*_15_ are continuous response variables, regression-type CARTs were considered. Consistently with the preceding OFAT and rule-based analyses, the laminar and turbulent datasets were treated independently. Consequently, four separate CART regression models were developed $T_{15}^{\mathrm{lam}},T_{15}^{\mathrm{turb}},p_{15}^{\mathrm{lam}},p_{15}^{\mathrm{turb}}$. This separation prevents the differences between the two flow regimes from being averaged within a single regression structure and enables the predictive relationships governing each response case to be evaluated independently.

In order to obtain a more formal and data-driven representation of the hydraulic system behavior, the CART method was applied to the dataset generated from the OFAT sensitivity analysis. The CART algorithm recursively partitions the parameter space into homogeneous regions according to the selected splitting criterion. Since both *T*_15_ and *p*_15_ are continuous variables, regression-type CARTs were considered.

**Figure 7:**
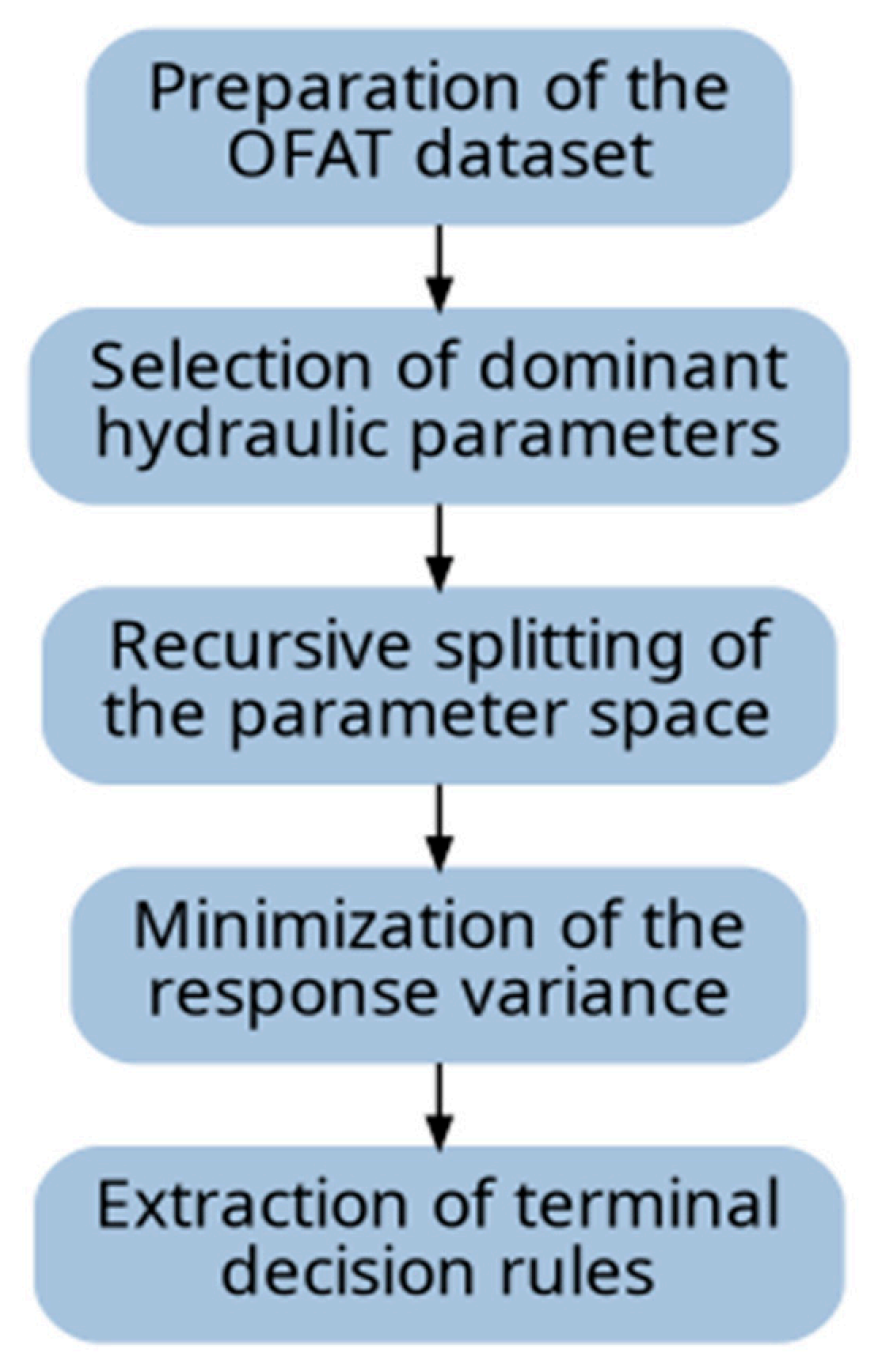
Flowchart for generating CART decision trees.

For a node *t*, the mean squared error is defined as:

(36) $$\mathrm{MSE}\left(t\right)=\frac{1}{N_{t}}\sum_{i\in t}(\left.y_{i}-{\bar{y}}_{t}{\left.)\right.}^{2}\right.$$

where *N_t_* is the number of samples in node *t*, *y_i_* denotes the observed response value, ${\bar{y}}_{t}$ is the mean response within node *t*. For each candidate split, the reduction in variance is evaluated as:

(37) $$\Delta \mathrm{MSE}=\mathrm{MSE}\left(t\right)-\left(\frac{N_{L}}{N_{t}}\mathrm{MSE}\left(t_{L}\right)+\frac{N_{R}}{N_{t}}\mathrm{MSE}\left(t_{R}\right)\right)$$

where *t_L_* and *t_R_* denote the left and right child nodes; *N_L_* and *N_R_* represent the number of samples in each subset. The optimal split is selected according to:

(38) $$x^{*}=\arg\underset{x_{i}}{\max}\Delta \mathrm{MSE}\left(x_{i}\right)$$

meaning that the parameter producing the largest variance reduction is selected as the splitting variable. The diagram in Figure 7 shows a flowchart for generating CART decision trees.

The CART regression analysis was based on the numerical observations obtained from the OFAT sensitivity analysis. Four independent regression trees were developed to represent the temporal and pressure responses under laminar and turbulent flow conditions. At each node, the splitting procedure selected the parameter and threshold providing the greatest reduction in the mean squared error, as described by Equations (36)–(38). The resulting trees provide piecewise-constant approximations of the relationships between the investigated hydraulic parameters and the corresponding response variables. Since the numerical dataset was generated using one-factor-at-a-time perturbations, the resulting tree structures should be interpreted primarily as exploratory representations of individual parameter effects within the investigated ranges, rather than as models demonstrating general predictive capability across arbitrary combinations of hydraulic parameters.

The CART analysis confirms that the transient hydraulic response may be effectively represented using a limited number of dominant parameters. The upper levels of the obtained trees are occupied by parameters exhibiting the highest OFAT sensitivity coefficients, demonstrating strong consistency between the sensitivity analysis and the machine learning model.

Following the qualitative rule-based analysis, quantitative CART regression models were constructed independently for each of the four response cases. Separate models were therefore developed for the temporal response under laminar flow $T_{15}^{\mathrm{lam}}$, the temporal response under turbulent flow $T_{15}^{\mathrm{turb}}$, the pressure response under laminar flow $p_{15}^{\mathrm{lam}}$, and the pressure response under turbulent flow $p_{15}^{\mathrm{turb}}$. This separation ensures that the regression trees preserve the response characteristics specific to each flow regime and prevents the averaging of laminar and turbulent behavior within a single model. The maximum tree depth was set to 3, and the minimum number of samples per leaf node was set to 2. These settings were intended to limit model complexity and preserve the interpretability of the resulting decision structures. No separate training and test subsets were created. Model performance was assessed using in-sample mean absolute error (MAE) and coefficient of determination *R*^2^ values, supplemented by leave-one-out cross-validation (LOOCV), with 28 observations used for training and one for validation in each of the 29 iterations.

#### 5.3.1. CART Model for the Temporal Response *T*_15_

**Figure A2:**
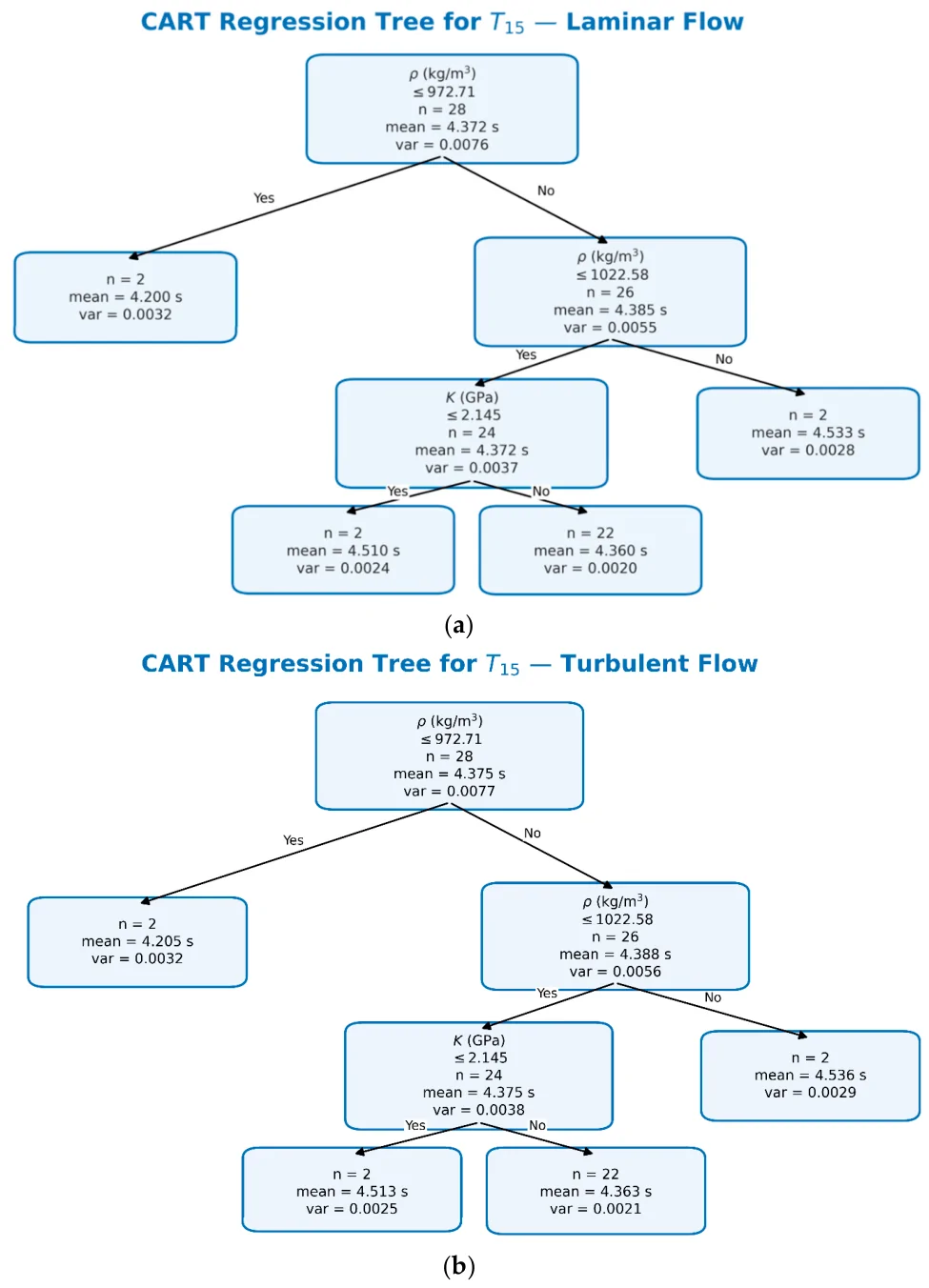

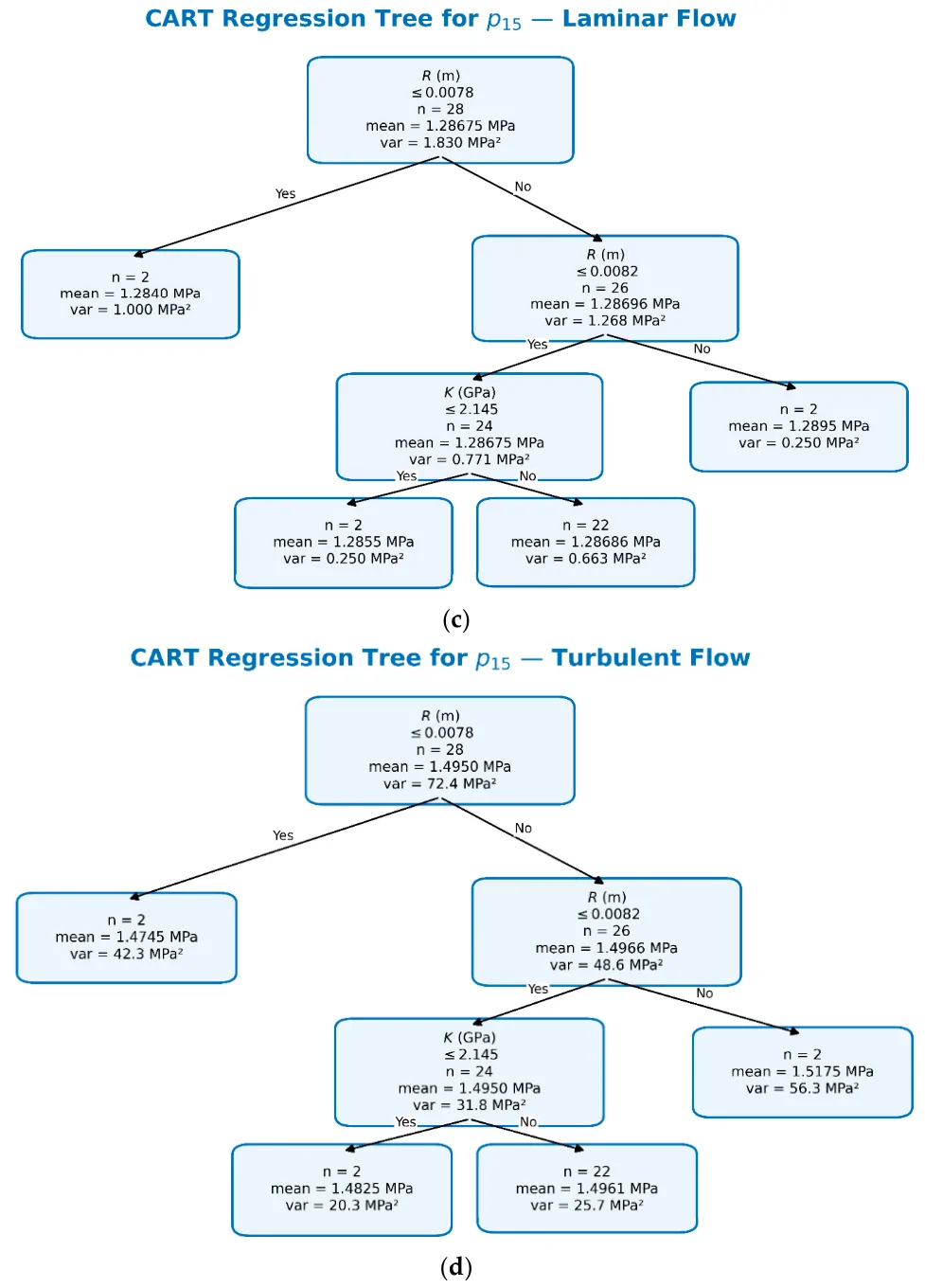
CART regression tree for the temporal response: (**a**) *T*_15_ under laminar flow conditions; (**b**) *T*_15_ under turbulent flow conditions; (**c**) *p*_15_ under laminar flow conditions; (**d**) *p*_15_ under turbulent flow conditions.

The CART regression models for *T*_15_ were developed independently using the OFAT observations corresponding to laminar and turbulent flow conditions. The original continuous values of *T*_15_ were used as the response variable, while the hydraulic parameters were introduced as predictors. The resulting regression trees are presented in Figure A2a for laminar flow and Figure A2b for turbulent flow.

For both flow regimes, fluid density *ρ* was identified as the primary splitting variable, while the bulk modulus *K* provided an additional refinement of the temporal-response prediction. This structure is consistent with the OFAT sensitivity analysis and the rule-based decision structures presented in Section 5.2, which identified *ρ* and *K* as the dominant parameters governing *T*_15_. The similarity of the two trees indicates that the principal parameter relationships governing the temporal response remain relatively stable between laminar and turbulent flow conditions.

Although the two models exhibit similar tree structures, they were constructed independently using their respective flow-regime datasets. This preserves the small differences in the response range and splitting thresholds associated with the two flow conditions without imposing a common regression structure.

#### 5.3.2. CART Model for the Pressure Response *p*_15_

The CART regression models for *p*_15_ were likewise developed independently for laminar and turbulent flow conditions using the corresponding OFAT observations. The original continuous pressure-response values were used as the response variable, and the hydraulic parameters were introduced as predictors. The resulting regression trees are presented in Figure A2c for laminar flow and Figure A2d for turbulent flow.

For both flow regimes, the pipe radius *R* was identified as the primary splitting variable, confirming its dominant influence on the pressure response established by the OFAT sensitivity analysis. The bulk modulus *K* provided additional refinement of the predictions, particularly within intermediate regions of the parameter space. This reveals a minor discrepancy with the OFAT results for the laminar pressure response, where viscosity *ν* was identified as the second most influential parameter. This difference reflects the distinct underlying mathematics of the two models. Therefore, combining both methods provides a more comprehensive and reliable characterization of parameter importance than relying on either method alone. However, the resulting tree structures support the qualitative relationships identified in the rule-based analysis, in which *R* provided the clearest separation of the pressure-response classes and *K* exhibited a more gradual influence.

The qualitative structure of the pressure-response models is therefore consistent across the two flow regimes, while the response ranges differ substantially. In particular, the turbulent-flow model represents a considerably wider range of *p*_15_ values than the laminar-flow model, consistent with the greater pressure sensitivity under turbulent conditions identified in the OFAT analysis.

#### 5.3.3. Performance of the CART Regression Models

The performance of the four independently developed CART regression models was evaluated using the mean absolute error (MAE) and coefficient of determination (*R*^2^). The MAE is defined as

(39) $$\mathrm{MAE}=\frac{1}{N}\sum_{i=1}^{N}|y_{i}-{\hat{y}}_{i}|$$

while the coefficient of determination is given by

(40) $$R^{2}=1-\frac{\sum_{i=1}^{N}{\left(y_{i}-{\hat{y}}_{i}\right)}^{2}}{\sum_{i=1}^{N}{\left(y_{i}-\bar{y}\right)}^{2}}$$

where *N* is the number of observations, *y_i_* is the observed response, ${\hat{y}}_{i}$ is the CART-predicted response, and $\bar{y}$ is the mean observed response. The resulting performance indicators are summarized in Table 8.

The performance indicators presented in Table 8 describe the reported degree of agreement between the CART regression models and the numerical responses obtained from the OFAT analysis. The coefficients of determination range from 0.657 to 0.717, indicating that the regression trees reproduce a substantial proportion of the response variability, although a noticeable proportion remains unexplained by the fitted models.

The temporal-response models exhibit similar MAE and *R*^2^ values under laminar and turbulent conditions. For the pressure-response models, the higher absolute MAE under turbulent conditions should be considered in relation to the wider range of pressure responses observed in this flow regime.

The CART models were developed primarily to identify dominant splitting variables, response thresholds, and interpretable parameter–response relationships. Consequently, the reported performance indicators should be interpreted as measures of model fit within the investigated OFAT dataset and should not, in the absence of documented independent validation, be regarded as evidence of predictive accuracy for previously unseen hydraulic configurations.

As an additional robustness check, LOOCV was performed for the four reconstructed CART models. The resulting cross-validated *R*^2^ values were 0.051 and 0.050 for the laminar and turbulent temporal-response models, respectively, and 0.028 and 0.115 for the corresponding pressure-response models. These results indicate limited predictive generalization within the available OFAT observations. Therefore, the CART models are primarily used to provide interpretable descriptions of dominant parameter–response relationships, rather than as independently validated predictive tools for arbitrary hydraulic configurations.

**Figure 8:**
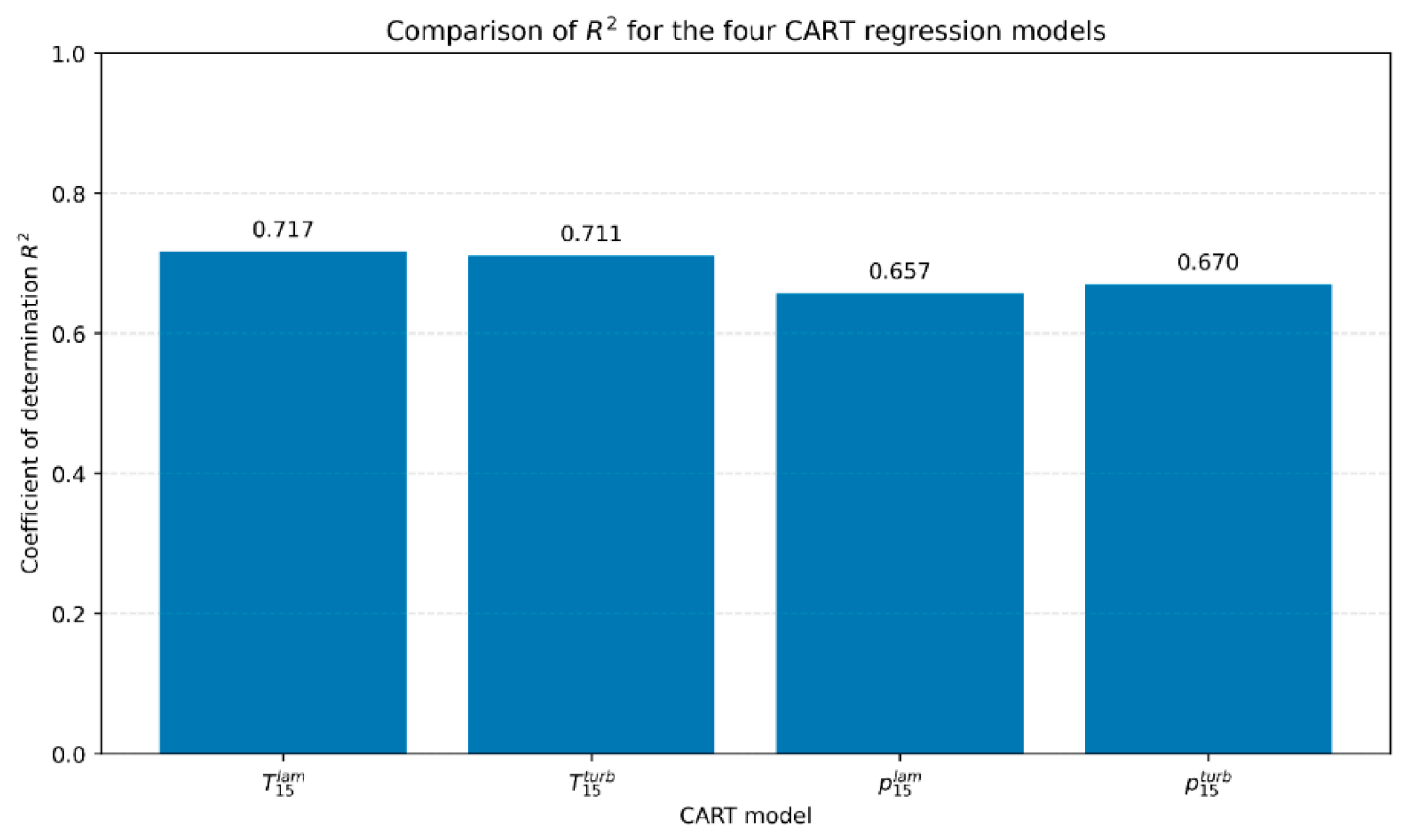
Comparison of the coefficient of determination *R*^2^ obtained for the four independently developed CART regression models.

As illustrated in Figure 8, the temporal-response models exhibit the highest *R*^2^ values, with only a small difference between laminar and turbulent flow. The pressure-response models show moderately lower *R*^2^ values, while the turbulent-pressure model performs marginally better than the laminar-pressure model according to this metric. Overall, the relatively small differences between the corresponding laminar and turbulent models indicate that independent treatment of the flow regimes preserves their specific response characteristics without substantially affecting the quality of the CART representation.

It should be emphasized that the obtained coefficients of determination, ranging from 0.657 to 0.717, indicate a moderate rather than high level of model fit. Although the CART models capture a substantial proportion of the response variability, approximately 28–34% remains unexplained within the evaluated data. Furthermore, the OFAT dataset comprises only 29 numerical configurations per flow regime, generated through individual variations in seven hydraulic parameters at four perturbation levels and the reference configuration. These characteristics limit the representation of the multidimensional parameter space and the assessment of more complex parameter relationships.

Consequently, the developed CART models should be regarded primarily as exploratory and interpretative tools rather than independently validated predictive models for engineering design or safety-critical water hammer applications. More comprehensive datasets and independent validation are required before their predictive reliability and generalization capability can be established.

#### 5.3.4. Overall Analytical Workflow

**Figure 9:**
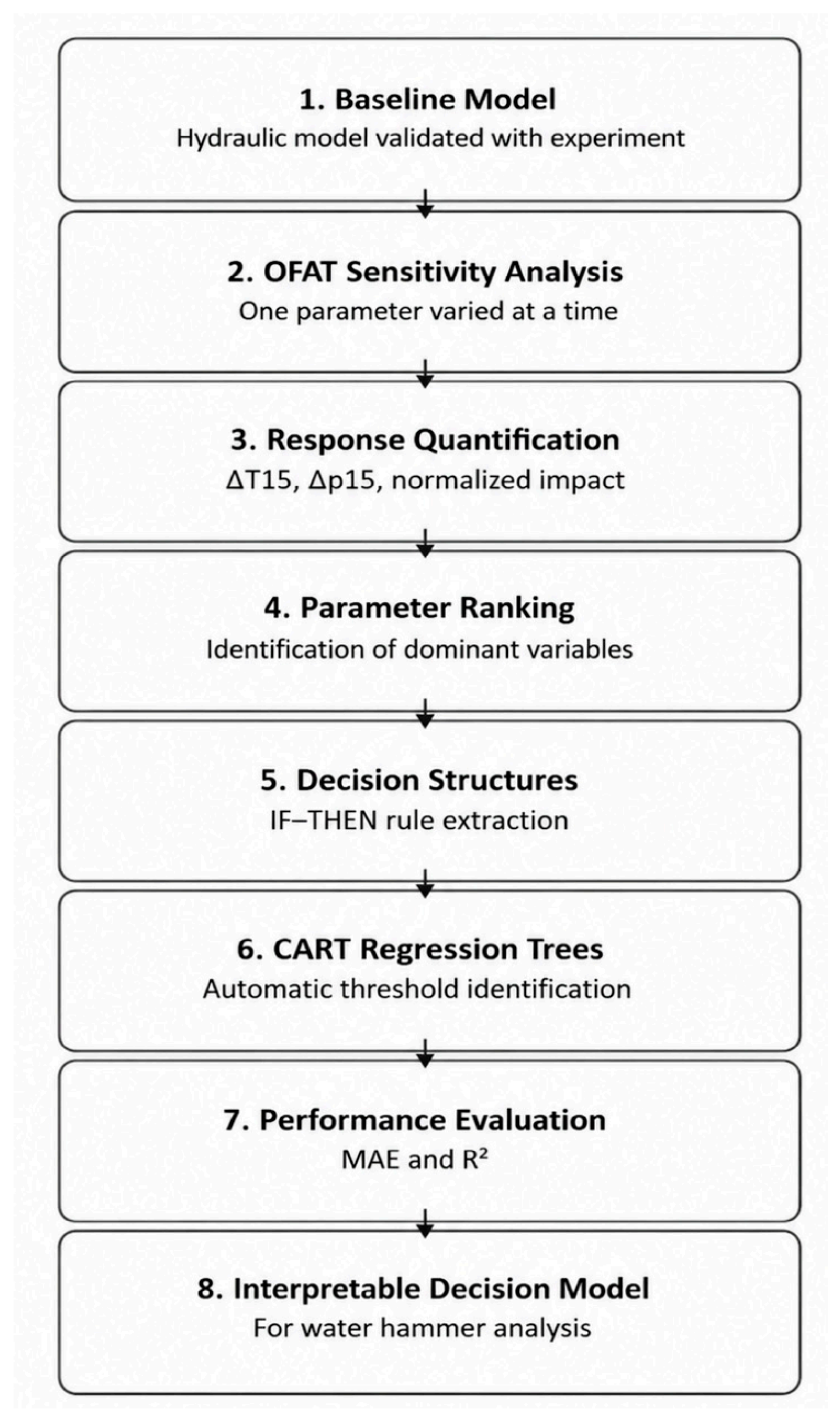
Overall workflow of the proposed OFAT decision-tree methodology for hydraulic system analysis under water hammer conditions.

The overall analytical workflow of the proposed methodology is presented in Figure 9. The procedure integrates hydraulic-model validation, OFAT sensitivity analysis, response quantification, parameter ranking, rule-based decision structures, and CART regression modeling. Both the rule-based structures and CART models were developed independently for laminar and turbulent flow conditions, resulting in four response cases: $T_{15}^{\mathrm{lam}}$, $T_{15}^{\mathrm{turb}}$, $p_{15}^{\mathrm{lam}}$, and $p_{15}^{\mathrm{turb}}$. The final stage comprises quantitative evaluation of the CART models using MAE and *R*^2^, followed by interpretation of the identified parameter thresholds and dominant parameter–response relationships.

## 6. Conclusions

This study quantified the influence of pipe material properties, pipe geometry, and liquid properties on water-hammer wave propagation and transient pressure response. A validated Method of Characteristics model with a convolution-based unsteady-friction formulation was combined with OFAT sensitivity analysis and interpretable CART models to distinguish the effects of pipe material, system geometry and liquid physical properties under laminar and turbulent conditions.

The present findings are consistent with recent studies highlighting the importance of pipe material and mechanical properties in water-hammer analysis. Kandil et al. [6] demonstrated experimentally and numerically that pipe rigidity and material properties influence transient pressure and frequency response, while Kadi-Rouabah et al. [7] investigated the sensitivity of overpressure and damping to pipeline characteristics. The present study complements these investigations by systematically comparing the effects of both liquid properties and pipe parameters on the time of the fifteenth pressure maximum *T*_15_, and its magnitude *p*_15_, under laminar and turbulent flow conditions. The results further demonstrate that parameter importance depends on the response quantity considered, highlighting the value of evaluating pressure amplitude and transient timing separately.The temporal structure of the water-hammer response is governed primarily by liquid density, liquid bulk modulus and pipe Young’s modulus. The ranking obtained for the investigated temporal response was

*ρ* > *K* > *E*_0_ > *e* > *R* > *ν_P_* > *ν*,

which remained essentially unchanged between laminar and turbulent flow. This indicates that the timing of successive pressure-wave amplitudes is controlled predominantly by the coupled compressibility of the liquid and pipe wall rather than by viscous effects.

The pressure response exhibited a different sensitivity pattern. Pipe radius was the dominant parameter in both flow regimes, whereas the importance of liquid bulk modulus and kinematic viscosity increased substantially under turbulent conditions. In particular, the normalized influence of *K* increased from 0.233% to 2.207%, while that of *ν* increased from 0.311% to 1.806%. These results indicate that the relative contribution of fluid properties to water-hammer pressure response depends on the flow regime.

The combined variation in pipe and liquid parameters produced pressure-wave celerity’s between approximately 1159 and 1479 m/s. The resulting difference of 320 m/s demonstrates that uncertainties in material properties, pipe geometry and liquid properties can substantially modify the predicted temporal structure of a water-hammer event.

From a materials-mechanics perspective, the results emphasize that Young’s modulus and Poisson’s ratio should not be treated merely as secondary input parameters. Through the pipe-wall compliance contribution to the pressure-wave celerity, they directly couple the mechanical behavior of the pipe material with the compressibility of the transported liquid.

The principal scientific contribution of this study is the integration of three complementary approaches for interpreting transient hydraulic responses: OFAT-based quantitative sensitivity assessment, qualitative rule-based decision structures, and CART regression-tree modeling. Their combined application provides a systematic means of comparing dominant parameter effects under laminar and turbulent flow conditions and translating numerical sensitivity results into interpretable decision rules and hierarchical regression representations. The proposed framework should be regarded as an exploratory and interpretative approach within the investigated parameter ranges rather than a comprehensive model of multiparameter interactions or an independently validated predictive tool.

The CART results should also be interpreted considering the limited dataset size and moderate model fit. With 29 numerical configurations per flow regime and coefficients of determination between 0.657 and 0.717, the developed regression trees provide useful qualitative insights into individual parameter effects but do not demonstrate sufficient predictive reliability for direct engineering applications. Further validation using larger datasets, including simultaneous variations in hydraulic parameters, is therefore necessary.

The OFAT and CART analyses provided complementary interpretations of the investigated parameter–response relationships. The resulting decision structures identified dominant variables and response thresholds within the investigated parameter space. However, because the underlying dataset was generated using one-factor-at-a-time perturbations, the present analysis does not fully resolve multiparameter interactions. Future work should therefore consider representative engineering pipe materials, temperature-dependent material and liquid properties, broader parameter ranges, and factorial or response-surface experimental designs.

## Figures and Tables

**Table 1:** Initial, minimal and maximal values of water hammer parameters.

Parameter	Laminar and Turbulent Case	Assumed Deviation	Minimal Value	Maximal Value
*ρ* [kg/m^3^]	997.65	±10%	*ρ*_1_ = 897.9	*ρ*_2_ = 1097.4
*R* [m]	0.008	±10%	*R*_1_ = 0.0072	*R*_2_ = 0.0088
*e* [m]	0.001 [m]	±10%	*e*_1_ = 0.0009	*e*_2_ = 0.0011
*ν_P_* [-]	0.35	±10%	*ν_P_*_1_ = 0.315	*ν_P_*_2_ = 0.385
*ν* [m^2^/s]	0.95 × 10^−6^	±10%	*ν*_1_ = 0.855 × 10^−6^	*ν*_2_ = 1.045 × 10^−6^
*K* [Pa]	2.2 × 10^9^	±10%	*K*_1_ = 1.98 × 10^9^	*K*_2_ = 2.42 × 10^9^
*E*_0_ [Pa]	124 × 10^9^	±10%	*E*_1_ = 111.6 × 10^9^	*E*_2_ = 136.4 × 10^9^

**Table 2:** Changes in pressure wave speed.

Parameter	*c* [m/s]	Parameter	*c* [m/s]	*Ξ* [-]	*α* [-]
*ρ*_1_	1382.3	*R*_1_	1324.2	14.516	1.008
*ρ*_2_	1250.3	*R*_2_	1298.8	17.314	0.984
*K*_1_	1258.0	*e*_1_	1297.5	17.469	0.983
*K*_2_	1360.5	*e*_2_	1323.0	14.643	1.007
*E*_1_	1295.6	*ν_P_*_1_	1308.8	16.195	1.012
*E*_2_	1324.7	*ν_P_*_2_	1314.2	15.597	0.975

**Table 3:** Single parameter effect from the qualitative analysis.

When: ↑ of	Then *p*_1_	and *p*_15_	and *T*_15_
*ρ*	↑	→ (very small change)	↑
*R*	↘	↑	↗
*e*	↗	→ (very small change)	↓
*ν_P_*	↗	→ (very small change)	↘
*ν*	→ (no change)	↓	→ (very small change)
*K*	↑	→ (very small change)	↓
*E*_0_	↑	→ (very small change)	↓

↑ denotes an increase, ↓ a decrease, ↗ a slight increase, and ↘ a slight decrease while → very small or unnoticed change of parameter.

**Table 4:** Response range and normalized impact coefficients for laminar and turbulent flows.

	Laminar Flow				Turbulent Flow			
Parameter	$R_{T_{15}}$ [s]	$I_{T_{15}}$ [%]	$R_{p_{15}}$ [Pa]	$I_{p_{15}}$ [%]	$R_{T_{15}}$ [s]	$I_{T_{15}}$ [%]	$R_{p_{15}}$ [Pa]	$I_{p_{15}}$ [%]
*ρ*	0.442	10.114	0	0.000	0.442	10.105	4000	0.268
*R*	0.063	1.442	7000	0.544	0.064	1.463	57,000	3.813
*e*	0.085	1.945	1000	0.078	0.085	1.943	8000	0.535
*ν_P_*	0.018	0.412	0	0.000	0.018	0.412	2000	0.134
*ν*	0.012	0.275	4000	0.311	0.009	0.206	27,000	1.806
*K*	0.348	7.963	3000	0.233	0.348	7.956	33,000	2.207
*E*	0.097	2.220	1000	0.078	0.098	2.241	10,000	0.669

**Table 5:** Structure of the dataset used for decision structure construction.

Flow Regime	Parameter	Perturbation	Parameter Value	*T*_15_ [s]	*p*_15_ [Pa]
Laminar	*ρ*	−10%	897.9	4.143	1.287 × 10^6^
Laminar	*ρ*	−5%	947.77	4.257	1.287 × 10^6^
Laminar	*ρ*	5%	1047.5	4.480	1.287 × 10^6^
Laminar	*ρ*	10%	1097.4	4.585	1.287 × 10^6^
…	…	…	…	…	…

**Table 6:** Classification of the temporal response of *T*_15_ for laminar and turbulent flow conditions.

		Laminar Flow		Turbulent Flow	
Parameter	Parameter Value	*T*_15_ [s]	Response Class	*T*_15_ [s]	Response Class
*ρ*	897.90 kg/m^3^	4.143	Short	4.148	Short
*ρ*	947.77 kg/m^3^	4.257	Short	4.261	Short
*ρ*	1047.50 kg/m^3^	4.480	Long	4.482	Long
*ρ*	1097.40 kg/m^3^	4.585	Long	4.590	Long
*K*	1.98 × 10^9^ Pa	4.558	Long	4.562	Long
*K*	2.09 × 10^9^ Pa	4.461	Long	4.463	Long
*K*	2.31 × 10^9^ Pa	4.288	Short	4.290	Short
*K*	2.42 × 10^9^ Pa	4.210	Short	4.214	Short
*E*	111.6 × 10^9^ Pa	4.423	Medium	4.428	Medium
*E*	117.8 × 10^9^ Pa	4.395	Medium	4.400	Medium
*E*	130.2 × 10^9^ Pa	4.347	Medium	4.351	Medium
*E*	136.4 × 10^9^ Pa	4.326	Medium	4.330	Medium

**Table 7:** Classification of the pressure response *p*_15_ under laminar and turbulent flow conditions.

		Laminar Flow		Turbulent Flow	
Parameter	Parameter Value	*p*_15_ [Pa]	Response Class	*p*_15_ [Pa]	Response Class
*R*	0.0072 m	1,283,000	Low	1,468,000	Low
*R*	0.0076 m	1,285,000	Low	1,481,000	Low
*R*	0.0084 m	1,289,000	High	1,510,000	High
*R*	0.0088 m	1,290,000	High	1,525,000	High
*ν*	0.855 × 10^6^ m^2^/s	1,289,000	High	1,510,000	High
*ν*	0.903 × 10^6^ m^2^/s	1,288,000	High	1,502,000	Medium
*ν*	0.998 × 10^6^ m^2^/s	1,286,000	Medium	1,489,000	Medium
*ν*	1.045 × 10^6^ m^2^/s	1,285,000	Low	1,483,000	Low
*K*	1.98 × 10^9^ Pa	1,285,000	Low	1,478,000	Low
*K*	2.09 × 10^9^ Pa	1,286,000	Medium	1,487,000	Medium
*K*	2.31 × 10^9^ Pa	1,287,000	Medium	1,503,000	Medium
*K*	2.42 × 10^9^ Pa	1,288,000	High	1,511,000	High

**Table 8:** In-sample performance indicators of the four independently developed CART regression models.

CART Model	MAE	*R* ^2^
$T_{15}^{\mathrm{lam}}$	0.0350 s	0.717
$T_{15}^{\mathrm{turb}}$	0.0356 s	0.711
$p_{15}^{\mathrm{lam}}$	584 Pa	0.657
$p_{15}^{\mathrm{turb}}$	4831 Pa	0.670

## Data Availability

The original contributions presented in this study are included in the article. Further inquiries can be directed to the corresponding author.

## Appendix A

**Table A1:** Results of the OFAT sensitivity analysis for individual hydraulic parameters under laminar and turbulent flow conditions.

Flow Regime	Para	Perturbation [%]	Parameter Value	*T*_15_ [s]	*p*_15_ [Pa]	Δ*T*_15_ [s]	Δ*T*_15_ [%]	Δ*p*_15_ [Pa]	Δ*p*_15_ [%]	dir (*T*_15_)	dir (*p*_15_)
lam	*ρ*	−10	897.9	4.143	1 × 10^6^	−0.227	−5.19450801	0	0	↓	≈
lam	*ρ*	−5	947.77	4.257	1 × 10^6^	−0.113	−2.58581236	0	0	↓	≈
lam	*ρ*	5	1047.5	4.48	1 × 10^6^	0.11	2.517162471	0	0	↑	≈
lam	*ρ*	10	1097.4	4.585	1 × 10^6^	0.215	4.919908467	0	0	↑	≈
lam	*R*	−10	0.0072	4.339	1 × 10^6^	−0.031	−0.70938215	−4000	−0.31080031	↓	↓
lam	*R*	−5	0.0076	4.356	1 × 10^6^	−0.014	−0.32036613	−2000	−0.15540016	↓	↓
lam	*R*	5	0.0084	4.386	1 × 10^6^	0.016	0.366132723	2000	0.155400155	↑	↑
lam	*R*	10	0.0088	4.402	1 × 10^6^	0.032	0.732265446	3000	0.233100233	↑	↑
lam	*e*	−10	0.0009	4.416	1 × 10^6^	0.046	1.052631579	−1000	−0.07770008	↑	≈
lam	*e*	−5	0.00095	4.392	1 × 10^6^	0.022	0.503432494	−1000	−0.07770008	↑	≈
lam	*e*	5	0.00105	4.35	1 × 10^6^	−0.02	−0.4576659	0	0	↓	≈
lam	*e*	10	0.0011	4.331	1 × 10^6^	−0.039	−0.89244851	0	0	↓	≈
lam	*ν_P_*	−10	0.315	4.378	1 × 10^6^	0.008	0.183066362	0	0	↑	≈
lam	*ν_P_*	−5	0.3325	4.374	1 × 10^6^	0.004	0.091533181	0	0	≈	≈
lam	*ν_P_*	5	0.3675	4.365	1 × 10^6^	−0.005	−0.11441648	0	0	↓	≈
lam	*ν_P_*	10	0.385	4.36	1 × 10^6^	−0.01	−0.22883295	0	0	↓	≈
lam	*ν*	−10	0.000000855	4.365	1 × 10^6^	−0.005	−0.11441648	2000	0.155400155	↓	↑
lam	*ν*	−5	0.000000903	4.367	1 × 10^6^	−0.003	−0.06864989	1000	0.077700078	≈	≈
lam	*ν*	5	0.000000998	4.372	1 × 10^6^	0.002	0.04576659	−1000	−0.07770008	≈	≈
lam	*ν*	10	0.000001045	4.377	1 × 10^6^	0.007	0.160183066	−2000	−0.15540016	↑	↓
lam	*K*	−10	1,980,000,000	4.558	1 × 10^6^	0.188	4.302059497	−2000	−0.15540016	↑	↓
lam	*K*	−5	2,090,000,000	4.461	1 × 10^6^	0.091	2.082379863	−1000	−0.07770008	↑	≈
lam	*K*	5	2,310,000,000	4.288	1 × 10^6^	−0.082	−1.87643021	0	0	↓	≈
lam	*K*	10	2,420,000,000	4.21	1 × 10^6^	−0.16	−3.66132723	1000	0.077700078	↓	≈
lam	*E*	−10	1.116 × 10^11^	4.423	1 × 10^6^	0.053	1.212814645	−1000	−0.07770008	↑	≈
lam	*E*	−5	1.178 × 10^11^	4.395	1 × 10^6^	0.025	0.57208238	−1000	−0.07770008	↑	≈
lam	*E*	5	1.302 × 10^11^	4.347	1 × 10^6^	−0.023	−0.52631579	0	0	↓	≈
lam	*E*	10	1.364 × 10^11^	4.326	1 × 10^6^	−0.044	−1.00686499	0	0	↓	≈
turb	*ρ*	−10	897.9	4.148	1 × 10^6^	−0.226	−5.16689529	−2000	−0.13377926	↓	↓
turb	*ρ*	−5	947.77	4.261	1 × 10^6^	−0.113	−2.58344765	−1000	−0.06688963	↓	≈
turb	*ρ*	5	1047.5	4.482	1 × 10^6^	0.108	2.469135802	1000	0.066889632	↑	≈
turb	*ρ*	10	1097.4	4.59	1 × 10^6^	0.216	4.938271605	2000	0.133779264	↑	↑
turb	*R*	−10	0.0072	4.343	1 × 10^6^	−0.031	−0.70873342	−27,000	−1.80602007	↓	↓
turb	*R*	−5	0.0076	4.358	1 × 10^6^	−0.016	−0.3657979	−14,000	−0.93645485	↓	↓
turb	*R*	5	0.0084	4.391	2 × 10^6^	0.017	0.388660265	15,000	1.003344482	↑	↑
turb	*R*	10	0.0088	4.407	2 × 10^6^	0.033	0.754458162	30,000	2.006688963	↑	↑
turb	*e*	−10	0.0009	4.421	1 × 10^6^	0.047	1.074531321	−4000	−0.26755853	↑	↓
turb	*e*	−5	0.00095	4.397	1 × 10^6^	0.023	0.525834476	−2000	−0.13377926	↑	↓
turb	*e*	5	0.00105	4.354	1 × 10^6^	−0.02	−0.45724737	2000	0.133779264	↓	↑
turb	*e*	10	0.0011	4.336	1 × 10^6^	−0.038	−0.86877	4000	0.267558528	↓	↑
turb	*ν_P_*	−10	0.315	4.383	1 × 10^6^	0.009	0.205761317	−1000	−0.06688963	↑	≈
turb	*ν_P_*	−5	0.3325	4.379	1 × 10^6^	0.005	0.114311843	0	0	↑	≈
turb	*ν_P_*	5	0.3675	4.37	1 × 10^6^	−0.004	−0.09144947	1000	0.066889632	≈	≈
turb	*ν_P_*	10	0.385	4.365	1 × 10^6^	−0.009	−0.20576132	1000	0.066889632	↓	≈
turb	*ν*	−10	0.000000855	4.37	2 × 10^6^	−0.004	−0.09144947	15,000	1.003344482	≈	↑
turb	*ν*	−5	0.000000903	4.372	2 × 10^6^	−0.002	−0.04572474	7000	0.468227425	≈	↑
turb	*ν*	5	0.000000998	4.377	1 × 10^6^	0.003	0.068587106	−6000	−0.40133779	≈	↓
turb	*ν*	10	0.000001045	4.379	1 × 10^6^	0.005	0.114311843	−12,000	−0.80267559	↑	↓
turb	*K*	−10	1,980,000,000	4.562	1 × 10^6^	0.188	4.298125286	−17,000	−1.13712375	↑	↓
turb	*K*	−5	2,090,000,000	4.463	1 × 10^6^	0.089	2.0347508	−8000	−0.53511706	↑	↓
turb	*K*	5	2,310,000,000	4.29	2 × 10^6^	−0.084	−1.92043896	8000	0.535117057	↓	↑
turb	*K*	10	2,420,000,000	4.214	2 × 10^6^	−0.16	−3.65797897	16,000	1.070234114	↓	↑
turb	*E*	−10	1.116 × 10^11^	4.428	1 × 10^6^	0.054	1.234567901	−5000	−0.33444816	↑	↓
turb	*E*	−5	1.178 × 10^11^	4.4	1 × 10^6^	0.026	0.594421582	−2000	−0.13377926	↑	↓
turb	*E*	5	1.302 × 10^11^	4.351	1 × 10^6^	−0.023	−0.52583448	2000	0.133779264	↓	↑
turb	*E*	10	1.364 × 10^11^	4.33	2 × 10^6^	−0.044	−1.00594422	5000	0.334448161	↓	↑

Note: ↑ indicates an increase, ↓ indicates a decrease, and ≈ indicates an approximately unchanged value of the corresponding response (*T*_15_ *or p*_15_) relative to the reference (baseline) OFAT configuration.

## References

[1] Plouraboué F. (2004). Review on water-hammer waves mechanical and theoretical foundations. Eur. J. Mech.-B/Fluids.

[2] Joukowsky N. (1900). Uber den hydraulischen Stoss in Wasserleitungsrohren (On the hydraulic hammer in water supply pipes). Mem. l’Academie Imp. Sci. St. Petersbourg.

[3] Tjuatja V., Keramat A., Pan B., Duan H.F., Brunone B., Meniconi S. (2023). Transient flow modeling in viscoelastic pipes: A comprehensive review of literature and analysis. Phys. Fluids.

[4] Simpson A.R., Wylie E.B. (1991). Large water-hammer pressures for column separation in pipelines. J. Hydraul. Eng..

[5] Kraiem D., Triki A. (2023). Tackling of hydraulic cavitation in pressurized pipe flow using high- or low-density polyethylene penstock and short-section. AQUA-Water Infrastruct. Ecosyst. Soc..

[6] Kandil M., El-Sayed T.A., Kamal A.M. (2024). Unveiling the impact of pipe materials on water hammer in pressure pipelines: An experimental and numerical study. Sci. Rep..

[7] Kadi-Rouabah L., Salah B., Zeroual A., Bounnah Y. (2026). Water hammer study in rigid and viscoelastic pipelines with sensitivity analysis of overpressure and damping. Int. J. Energy Water Resour..

[8] Henclik S. (2018). Analytical solution and numerical study on water hammer in a pipeline closed with an elastically attached valve. J. Sound Vib..

[9] Henclik S. (2018). Numerical modeling of water hammer with fluid–structure interaction in a pipeline with viscoelastic supports. J. Fluids Struct..

[10] Tijsseling A.S. (2007). Water hammer with fluid–structure interaction in thick-walled pipes. Comput. Struct..

[11] Wylie E.B., Streeter V.L., Suo L. (1993). Fluid Transients in Systems.

[12] Bergant A., Tijsseling A.S., Vítkovský J.P., Covas D.I.C., Simpson A.R., Lambert M.F. (2008). Parameters affecting water-hammer wave attenuation, shape and timing—Part 1: Mathematical tools. J. Hydraul. Res..

[13] Roberts A., Thomas B., Sewell P., Hoare E. (2019). Generating renewable power from water hammer pressure surges. Renew. Energy.

[14] Suzuki K. (1990). A New Hydraulic Pressure Intensifier Using Oil Hammer. J. Fluids Eng..

[15] Zha C., Pan C., Sun Z., Liu Q. (2024). Reliability sensitivity analysis for water hammer-induced stress failure of fluid-conveying pipe. Appl. Math. Model..

[16] Goudar C.T., Sonnad J.R. (2008). Comparison of the iterative approximations of the Colebrook-White equation. Hydrocarb. Process..

[17] Warda H.A., Wahba E.M., Salah El-Din M. (2020). Computational Fluid Dynamics (CFD) simulation of liquid column separation in pipe transients. Alex. Eng. J..

[18] Urbanowicz K., Bergant A., Stosiak M., Deptuła A., Karpenko M., Kubrak M., Kodura A. (2022). Water Hammer Simulation Using Simplified Convolution-Based Unsteady Friction Model. Water.

[19] Urbanowicz K. (2017). Analytical expressions for effective weighting functions used during simulations of water hammer. J. Theor. Appl. Mech..

[20] Adamkowski A., Lewandowski M. (2006). Experimental Examination of Unsteady Friction Models for Transient Pipe Flow Simulation. J. Fluids Eng..

[21] Miller M.D., Thornton M.A. (2007). Multiple-Valued Logic: Concepts and Representations.

[22] Wafi D., Rahman M. (2017). A new approach for multi-valued computing using machine learning. Proceedings of the 2017 IEEE International Conference on Rebooting Computing (ICRC), Washington, DC, USA, 8–9 November 2017.

[23] Garribba S., Guagnini E., Mussio P. (2009). Multiple-valued logic trees: Meaning and prime implicants. IEEE Trans. Reliab..

[24] Gaudet V., Butler J.T., Wille R., Homma N. (2016). Guest Editorial Emerging Topics in Multiple-Valued Logic and Its Applications. IEEE J. Emerg. Sel. Top. Circuits Syst..

[25] Smith K.C. (1981). The prospects for multivalued logic: A technology and applications view. IEEE Trans. Comput..

[26] Smith K.C. (1988). A Multiple Valued Logic: A Tutorial and Appreciation. Computer.

[27] Sheikholeslami A., Yoshimura R., Gulak P.G. (1998). Look-up tables (LUTs) for multiple-valued, combinational logic. Proceedings of the 28th IEEE International Symposium on Multiple-Valued Logic (Cat. No. 98CB36138), Fukuoka, Japan, 29 May 1998.

[28] Drechsler R. (1996). Verification of Multi-Valued Logic Networks using OMDDs. Proceedings of the 26th IEEE International Symposium on Multiple-Valued Logic (ISMVL’96), Santiago de Compostela, Spain, 29–31 January 1996.

[29] Liang J., Chen L., Han J., Lombardi F. (2014). Design and Evaluation of Multiple Valued Logic Gates Using Pseudo N-Type Carbon Nanotube FETs. IEEE Trans. Nanotechnol..

[30] Drechsler R., Thornton M., Wessels D. (2000). MDD-based synthesis of multi-valued logic networks. Proceedings of the 30th IEEE International Symposium on Multiple-Valued Logic (ISMVL 2000), Portland, OR, USA, 23–25 May 2000.

[31] Waho T. (1995). Resonant tunneling transistor and its application to multiple-valued logic circuits. Proceedings of the 25th International Symposium on Multiple-Valued Logic, Bloomington, IN, USA, 23–25 May 1995.

[32] Current K.W. (2008). Multiple-Valued Logic Circuits. Digital Design and Fabrication.

[33] Zilic Z., Vranesic Z.G. (1993). Multiple-valued logic in FPGAs. Proceedings of the 36th Midwest Symposium on Circuits and Systems, Detroit, MI, USA, 16–18 August 1993.

[34] Pierzchala E., Perkowski M.A., Grygiel S. (1994). A field programmable analog array for continuous, fuzzy, and multi-valued logic application. Proceedings of the 24th International Symposium on Multiple-Valued Logic (ISMVL’94), Boston, MA, USA, 25–27 May 1994.

[35] Maharsi D.A., Ta’dung J.C., Jannoke L., Budi I.S. (2022). Sensitivity analysis using One-Factor-at-a-Time and Response Surface Method in liquid dominated geothermal reservoir. PETRO J. Ilm. Tek. Perminyakan.

[36] Delgarm N., Sajadi B., Azarbad K., Delgarm S. (2018). Sensitivity analysis of building energy performance: A simulation-based approach using OFAT and variance-based sensitivity analysis methods. J. Build. Eng..

[37] Gey S., Nedelec E. (2005). Model selection for CART regression trees. IEEE Trans. Inf. Theory.

